# Time Scales of Gaseous Smoke Contamination Indoors from Real and Simulated Wildland-Urban Interface Fires

**DOI:** 10.1021/acsestair.5c00358

**Published:** 2025-12-05

**Authors:** Michael F. Link, Aika Y. Davis, Nathan M. Lima, Ryan L. Falkenstein-Smith, Rileigh L. Robertson, Thomas G. Cleary, Steven Emmerich, Dustin Poppendieck

**Affiliations:** 10833 National Institute of Standards and Technology, Gaithersburg, Maryland 20899, United States

**Keywords:** wildland–urban interface, indoor air quality, nonmethane organic gases, proton-transfer reaction mass spectrometry, smoke contamination

## Abstract

Fires occurring at the wildland-urban interface (WUI) can produce smoke, that contains unique chemicals from the combustion of urban structures, which can then contaminate nearby buildings and affect indoor air quality. Assessing property loss and possible occupant exposure to persistent contamination from WUI smoke is challenging, in part because of a lack of measurements detailing chemical contamination in real indoor environments after WUI events. Here, we mimic contamination from a WUI fire by repeatedly exposing a test house to smoke from combustion of residential building surrogates and measure the persistence of volatile nonmethane organic gas (NMOG) contamination. Over the 1.5 month experimental period, we observed an increase in emission rates of 31 NMOGs, indicating the formation of surface reservoirs indoors that increase with subsequent burns. We observe off-gassing time scales of less than 10 days for many highly volatile NMOGs like acetonitrile, acrylonitrile, and styrene. Other NMOGs, like naphthalene and C12 aromatics, took longer than 10 days to off-gas and show emissions persistently elevated above background for at least three months after the end of the experiments. The NMOG emissions from contamination in the test house were lower when compared with a house affected by the Marshall Fire in Colorado. However, the NMOG off-gassing times measured in the test house were longer.

## Graphical abstract

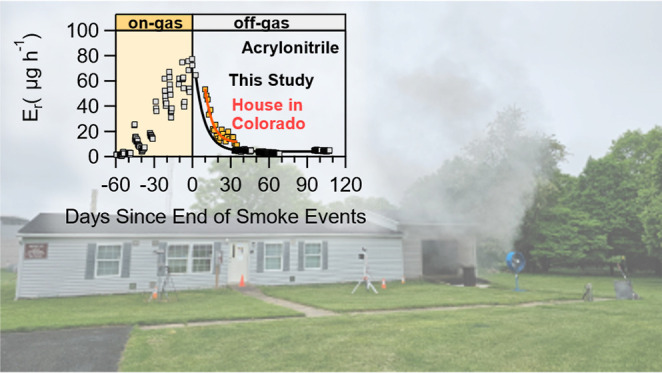

## Introduction

Fires occurring at the wildland-urban interface (WUI) can produce smoke from combustion of urban materials, like cars and structures, that can enter buildings and negatively impact indoor air quality.^1–3^ High-profile fires occurring at the WUI have prompted research increasingly focused on contaminants in the smoke and their fate as they impact both outdoor and indoor air quality.^4,5^ In one example, studies of the 2021 Marshall Fire in Colorado demonstrated that both gaseous and particulate contaminants in smoke contaminated nearby homes, with occupants reporting health symptoms, possibly from exposure to contaminants.^1–3^

Understanding the risk of occupant exposure to WUI fire smoke in contaminated homes is critical as the smoke is expected to be enriched in toxic compounds (e.g., polycyclic aromatic hydrocarbons, hydrochloric acid, and hydrogen cyanide) compared to wildfire smoke.^6,7^ Despite the increased attention on WUI fires, limited research is available showing how long gaseous smoke constituents that have entered a home can continue to affect indoor air quality after a fire is extinguished.^2,8^ Consequently, providing practical advice to homeowners describing when, or if, it could be safe to re-enter and inhabit a smoke-contaminated home is limited by a lack of understanding of how long a home may stay contaminated at unhealthy levels.^9^ Guidance documents such as those recently released by ASHRAE^10^ and the Underwriters Laboratory, UL (https://wildfirehealthrisks.org/wp-content/uploads/2024/10/UL200C_Public-Health-Impacts-of-WUI-Fires.pdf), on preparation and protection from WUI and wildfire smoke could benefit from an additional understanding of smoke contamination in real indoor environments. We do not address the question of whether it is safe to inhabit a home contaminated by WUI fire smoke in this current study but instead focus on quantifying how long indoor air might be expected to be impacted by smoke contamination.

Nonmethane organic gases (NMOGs) are a class of volatile contaminants partially responsible for the odors associated with smoke contamination. Odor is one of the key metrics used in property insurance assessments to assess physical damage or loss from smoke or noxious gas contamination.^11^ NMOGs can remain at elevated concentrations in indoor air for extended periods (i.e., days to months) after contamination, the persistence of which will depend on their chemical properties, outdoor air ventilation rates of the building, and surface properties that affect sorption kinetics in the indoor environment.^12–14^ However, little is known about how much NMOG contamination can occur indoors during a typical WUI fire event and how long that contamination may persist and impact indoor air quality.^9,15,16^

There are several key challenges to understanding the extent of NMOG contamination from WUI fires and persistence time scales of gaseous contaminants. First, in situ measurements are needed to understand the fast and slow processes associated with smoke contamination indoors, but deploying measurements in buildings affected by real WUI fires quickly for this purpose requires coordination among measurement personnel, emergency responders, and community members. Second, background concentrations of NMOGs are typically much higher (e.g., an order of magnitude or more) indoors than outdoors, and thus, differentiating between smoke contaminants and background NMOGs may be difficult.^2,16,17^ Real-time mass spectrometry measurements can be useful for measuring NMOG contamination but can also be complicated by the variety of chemicals present in indoor air that can interfere with the quantification of target NMOGs.^18^ Third, deposited contaminants may re-emit from surfaces and persist indoors on varying time scales ranging from hours to years.^19–22^ Experimental designs that can simulate realistic indoor contamination from WUI fire smoke are needed to address the key challenges identified here to better understand how to provide guidance for health and assess the effectiveness of remediation methods.

We performed experiments contaminating a test house with surrogate WUI fire smoke to understand how much NMOG contamination could be expected to occur from a WUI fire and how long it might take for NMOG decontamination via ventilation, in the absence of remediation actions. Recently, Link, Davis et al. (2025) quantified yields of NMOGs emitted from small mixed fuel cribs, designed to be surrogates for residential buildings, under a 0.5 MW hood calorimeter.^23^ We combusted the same surrogates outside of our test house to simulate the intrusion of WUI fire smoke indoors and subsequent contamination of the indoor environment. We used the NMOG smoke signatures measured from the laboratory experiments to identify NMOGs, distinct from the background, that contaminated the house. We then quantified NMOG emissions from contamination in the test house and compared them to reported emissions measured in a house in Colorado affected by the Marshall Fire.

## Methods and Materials

### Manufactured Test House

The National Institute of Standards and Technology (NIST) manufactured test house (called “test house” from here), located on the NIST Gaithersburg, MD campus, was constructed in 2002 and retrofitted multiple times over multiple years to increase building airtightness.^24^ A simplified diagram of the test house is shown in Figure S1. Among other research activities, the test house had been used to measure the transport of emissions from portable generators for nearly a decade.^25^ Thus, background contaminant emissions observed in this study may be influenced by persistent emissions from deposited contaminants resulting from the generator experiments. The test house temperature was controlled via the central HVAC system to 24 ± 1 °C. All doors to rooms inside the house were kept open except for 2 weeks in May when a bedroom door was shut. Do-it-yourself air cleaners were deployed in various configurations in April and May but were not observed to affect NMOG concentrations. Three months after the smoke contamination experiments ended, we removed all of the carpet in the test house to measure the effects on the NMOG emissions.

### Air Change Rate Measurements

During our experiments, the outdoor air change rate (λ) was measured via tracer decay tests using SF_6_. For most of the measurement campaign, SF_6_ was injected every 6 h into the central air intake and distributed throughout the test house via supply ducts. To account for λ measurement interruptions (e.g., loss of power to the injection system, malfunctioning injection, or instrument problems) we experienced during the campaign, we parametrized the λ from available SF_6_ measurements using a polynomial fit of λ to time (Figure S2) for use in NMOG emission rate calculations. We note that the polynomial fit to time is only empirical and does not reflect the impacts of differences in indoor versus outdoor temperature, wind speed/direction, ventilation, or other house operations that are known to influence λ. The average λ from April to September was 0.19 h^–1^ ± 0.06 h^–1^ (2σ). We show additional analyses of the λ parametrization (Figure S3) and consequent impacts on calculated NMOG emissions (Figure S4) in the Supporting Information. We also calculated λ values using available measurements of methane (CH_4_) emissions^26^ and found reasonable agreement (average ratio of SF_6_ to CH_4_ λ values = 1.01 ± 0.26) with SF_6_-derived λ values (Figure S5).

### Smoke Generation and Test House Contamination Methods

We generated smoke from combustion of mixed-fuel cribs designed to be surrogates for residential buildings (hereafter called “surrogates”).^23,27^ Information on surrogate design and combustion properties can be found in Davis et al. (2025). Briefly, surrogates were constructed by using a rectangular array of sticks of differing composition. Approximately half of the mass of the surrogate was noncombustible gypsum. The other half of the total mass was combustible and composed of approximately 70% wood (spruce-pine-fir and oriented strand board) and approximately 30% synthetic polymers [polyurethane rubber, polyvinyl chloride (PVC), and acrylonitrile-butadiene-styrene plastic]. The small, low-density surrogates combusted in this study weighed approximately 6 kg with dimensions of 30.0 cm × 30.0 cm × 28.6 cm.^23^

Measurements of smoke contamination were performed at the test house from April to mid-September 2024. Instruments were moved from the test house for the month of June to perform the measurements published by Link, Davis et al. (2025). Measurements resumed in the test house in early July. From April 15 to May 30, 2024, 11 surrogate burns occurred outside the test house to generate smoke. Two preliminary burns were performed prior to the first surrogate burn of the experimental set we consider here, but the smoke introduction and house operation methods were not optimized, and thus we consider the time between April 4th and the beginning of the first burn on April 15th to represent “background” conditions. Although not a perfect replication of the dynamics of a real WUI fire, the experimental design of burning surrogates in 11 individual experiments instead of one larger burn allowed us to easily control the burn experiment time and understand what emissions were entering the home based on previous characterizations^23^ and required less time and logistical demands to perform.

Surrogate burns typically occurred around 8:30 a.m. on days with low wind speeds (e.g., <8 km h^–1^). Surrogates were placed inside an open-ended, large (approximately 200 L) steel drum outside the test house’s garage on a small platform. Surrogates were ignited with small packages of the ignition fuel. A large fan was used to direct smoke into the open garage. Immediately prior to ignition, the door between the garage and living room/kitchen was opened 7 mm, which allowed smoke directed into the garage by the fan to enter the test house. We note that the smoke introduction method used in our experiments may not represent smoke infiltration in a sealed home contaminated with smoke from a real WUI fire. Burn experiments typically lasted 45 min, after which all exterior doors to the test house were closed. No entry to the test house was permitted until at least 48 h after a burn. Other times, entry to the test house was limited to short periods for instrument maintenance.

### Proton-Transfer Reaction Mass Spectrometer

We used time-of-flight proton-transfer reaction mass spectrometer (PTR-MS) to measure NMOGs in the test house. The PTR-MS alternately sampled from three different locations (outside, a bedroom, and the kitchen) every 4 min. The sampling point for the outside was located approximately 4 m from the front of the test house and elevated to 2 m above the ground. A pump pulled air through a 9.53 mm O.D. (7.95 mm I.D.) Perfluoroalkoxy alkane (PFA) tubing at a flow rate of 15 L min^–1^, and the PTR-MS subsampled the main flow orthogonally via a PFA tee at a flow rate of 100 mL min^–1^. Sampling lines not actively sampled by PTR-MS were flushed with 15 L min^–1^ of sample air using a supplemental bypass pump. A 10 μm polytetrafluoroethylene (PTFE) filter was installed at the end of each sample line to filter particles from the PTR-MS sample air. The filter was replaced approximately 48 h after every burn. Select samples were collected on multibed sorbent tubes and subsequently analyzed with the PTR-MS using a coupled thermal-desorption gas chromatograph (GC-PTR-MS).

We performed hourly five-point calibrations (including instrument zeros with ultrapure zero air) with three different multicomponent NMOG cylinders for the 5 months the PTR-MS was sampling from the test house (except for several week-long interruptions). We directly calibrated for over 30 NMOGs and observed sensitivities (ion counts per second per nmol per mol) to vary by no more than 15% between April and May. After the PTR-MS left the test house at the beginning of June and returned in July, we observed an increase of approximately 20% for all sensitivities of NMOGs being calibrated at that time. We used a NMOG-specific median sensitivity for the April and May period and an increased median sensitivity value for the post-July period for the conversion of the instrument signal to the mole fraction. We applied a sensitivity determined from a relationship of the H_3_O^+^ ion–molecule rate constant (k_PTR_) versus sensitivity (determined for our instrument as reported in a previous study^28^) for NMOGs we did not directly calibrate.

We report NMOG measurements in units of mole fraction (mol of NMOG per mole of air). As described in Link, Davis et al. (2025), we measured yields of 201 NMOGs from combustion of the surrogates under a hood calorimeter. We used these measurements of NMOGs from the calorimeter experiments to constrain what NMOGs we considered when assessing test house contamination. Of the 201 NMOGs measured from the hood calorimeter experiments, we quantified 31 as persistent (i.e., emissions elevated above background at the end of all of the burn experiments) NMOG contaminants in the test house.

We categorized the 31 NMOGs we quantified from the test house based on how confidently we could identify NMOGs from their ion formulas. Our categorization method was informed by the recent study of ion interferences by Zhang et al. (2025).^36^ Using several GC-PTR-MS measurements, we identified whether an ion formula likely contained isomeric contributions or product ion interferences. We characterized ion formula with a single, identifiable (based on retention times) NMOG contributing to the selected ion chromatogram as category I compounds, meaning that we report the identity and quantified emission rates of these NMOGs with high confidence (example in Figure S6). Category II compounds had multiple isomers contributing to an ion formula (Figure S7). Category III compounds could also have product ion interferences in the chromatogram in addition to isomers (Figure S8). Uncertainty in the quantification of category II NMOGs arises from possible differences in sensitivities between isomers. Uncertainty in the quantification of category III NMOGs additionally arises from product ion interferences. We characterized a total of 9, 13, and 9 NMOGs as category I, II, and III compounds, respectively (Table S1). Additional details of this analysis and surrogate compounds used to determine vapor pressure and octanol–air partitioning coefficients^29^ (Table S2) can be found in the Supporting Information.

### Whole-House Emission Rates

To quantify the strength of the smoke contamination source in the test house, we calculate effective whole-house NMOG emission rates (*E*_r_), as shown in eq 1

1 $$E_{r}=\left(C_{in}-C_{out}\right)\cdot V\cdot \lambda$$

where *E*_r_ is in μg h^–1^, *C*_in_ and *C*_out_ are the indoor and outdoor concentrations of NMOGs (μg m^–3^), *V* is the volume of the test house (324 m^3^),^26^ and λ is the outdoor air change rate (h^–1^). NMOG mass concentrations were calculated via conversion of mole fractions to NMOG number densities at 25 °C and 100 kPa for the test house and 25 °C and 85 kPa for the measurements from Dresser et al.^2^ (study performed in Colorado at 1650 m elevation). These *E*_r_ calculations are similar to those employed by other studies.^30–32^ We specify that these *E*_r_ values are effective because they do not account for factors such as changes in temperature, nonuniform indoor concentrations, or chemical reactions that may act as sources or sinks. We excluded mole fraction data that were collected 48 h after each burn experiment from our NMOG *E*_r_ calculations to allow uniform mixing of the contaminants throughout the test house.

## Results and Discussion

### Transient and Persistent Increases in Indoor NMOGs from Smoke

Figure 1 shows the mole fractions of two example NMOGs, acrylonitrile and naphthalene, that were produced with high yields (relative to other NMOGs) from combustion of the surrogates under a hood calorimeter in a previous study. In response to smoke introductions to the test house, we observed orders-of-magnitude mole fraction increases of NMOGs originating from combustion of the surrogate (Figure 1A). After the burns, these peak mole fractions decreased over tens of hours (48 h immediately after smoke events shown by lightly colored traces in Figure 1A) to values that were elevated above background (background shown by a yellow shaded region in Figure 1A).

**Figure 1:**
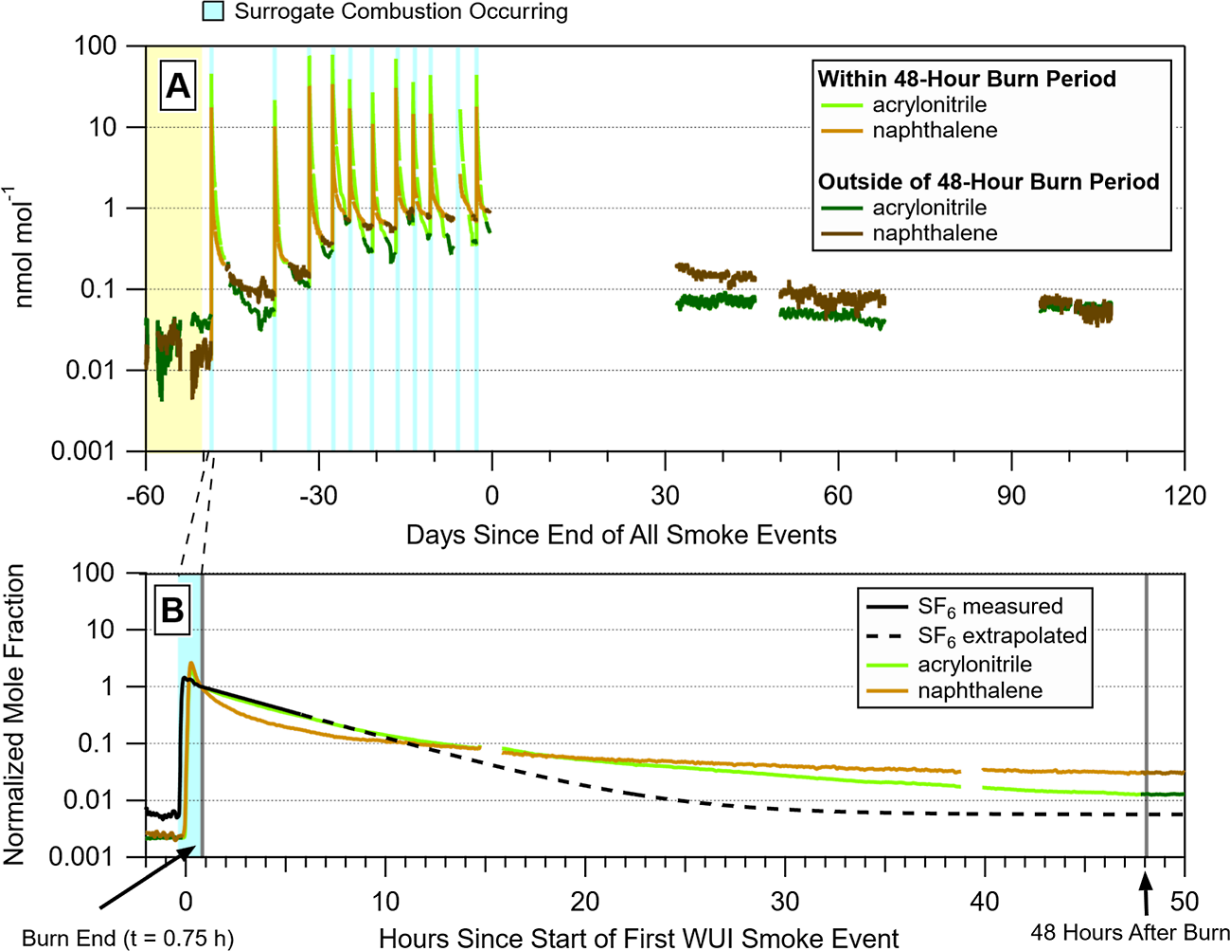
(A) Mole fraction time series for acrylonitrile and naphthalene over the six-month measurement period. Data from the 48 h around two preliminary burns performed on April 4 (day −59) and April 9 (day −54) are removed to clearly show the “background” period (shaded yellow area). Blue shaded areas show when a surrogate was combusted outside of the test house and correspond to an individual burn experiment/smoke event. Gaps in data are from when the PTR-MS was not sampling. (B) Normalized mole fraction time series of acrylonitrile (green), naphthalene (brown), and SF_6_ (black) measured during the first surrogate burn (shaded blue area). The arrows on the bottom of the panel indicate the bounds of when the “48 h after start of burn period” was defined for each surrogate burn. SF_6_ was measured for only 6 h before another injection increased concentrations during this burn, and thus we extrapolated from the initial injection in the figure.

As a result of contamination from repeated smoke events, the test house emission source of both acrylonitrile and naphthalene increased, resulting in indoor air concentrations persistently above background (shown by darker colored sections of NMOG tracers in Figure 1A). For example, prior to the start of the first surrogate burn (55 days before the end of the smoke events), average mole fractions (average of 1 day ± 2σ) of acrylonitrile were at 28 pmol mol^–1^ (±13 pmol mol^–1^) and were at 20 pmol mol^–1^ (±6 pmol mol^–1^) for naphthalene. In comparison, 35 days after the end of the smoke events, average mole fractions were approximately two times higher at 68 pmol mol^–1^ (±5 pmol mol^–1^) for acrylonitrile and eight times higher at 170 pmol mol^–1^ (±20 pmol mol^–1^) for naphthalene. Further, 100 days after the end of the experiments, average mole fractions for both NMOGs were still elevated above the background at approximately 60 pmol mol^–1^.

Figure 1B shows NMOG mole fractions for a 50 h period encompassing the first surrogate burn. During the 45 min burn (shown by the light blue shading), NMOGs enter the house and increase mole fractions several orders of magnitude above background concentrations. After the smoke event has ended, NMOG mole fractions decrease at different rates. We describe the rates of NMOG mole fraction decrease shown in Figure 1B by their mole fraction decay time, which is the time it takes for a NMOG to decrease to 1/*e* (approximately 37%) of its initial mole fraction, assuming a first-order decay. A smaller decay time indicates a faster decrease in NMOG mole fractions.

For the burn experiment shown in Figure 1B, the decay time of SF_6_ (black trace) injected prior to the burn experiment is 4.4 h and defines λ (the SF_6_ decay time is the inverse of λ or 0.23 h^–1^ ± 0.01 h^–1^) for the test house during the experiment. In the first 5 h after the burn, the decay of acrylonitrile is only slightly faster than SF_6_ (decay time = 3.9 h), whereas the decay of naphthalene is faster (decay time = 1.3 h). Consistent with our observations, Li et al. (2023)^19^ observed that oxygenated NMOGs from simulated wildfire smoke rapidly decreased in concentration (after a pulsed injection) compared to SF_6_. The authors demonstrated that NMOGs can undergo rapid deposition to surfaces, achieve a temporary equilibrium between adsorption and desorption on surfaces, and then produce elevated indoor air concentrations driven by surface desorption. In the approximately 10 h after the burn event, both acrylonitrile and naphthalene mole fractions typically decrease slowly. They then remain elevated by an order of magnitude above preburn background 48 h after the burn. Our observations of faster NMOG mole fraction decays, compared to SF_6_, point to NMOG deposition that creates a surface contamination emission source that, in turn, creates persistently high NMOG mole fractions indoors.

### NMOG Emission Rates Show Periods of On-Gassing and Off-Gassing

In the two months that burn experiments were performed, we observed an increase in the whole house emission rates (*E*_r_) of NMOGs originating from combustion of the surrogates, a period which we refer to as “on-gassing” (Figure 2, orange shaded area). We focus our analysis on 31 NMOGs whose *E*_r_ at the end of the on-gassing period was at least 5 μg h^–1^ above the background. Figure 2 shows four example NMOGs that showed on-gassing. The gray markers in Figure 2 show 6 h average *E*_r_ calculated outside of the 48 h immediately after each burn. Colored markers in Figure 2 show the average *E*_r_ values on select days (background, day 2, day 35, day 60, day 98, and day 105) to highlight specific times that are useful for discussing off-gassing time scales of NMOGs. The time points indicated by these markers will be the focus of the rest of the analysis and discussions in later sections of the manuscript. The star markers show the “background” *E*_r_ defined as the average (±2.5 days) *E*_r_ measured 55 days before the end of the burn experiments. Table S3 summarizes average *E*_r_ values at time points indicated by the colored markers in Figure 2 for the 31 NMOGs highlighted in this study.

**Figure 2:**
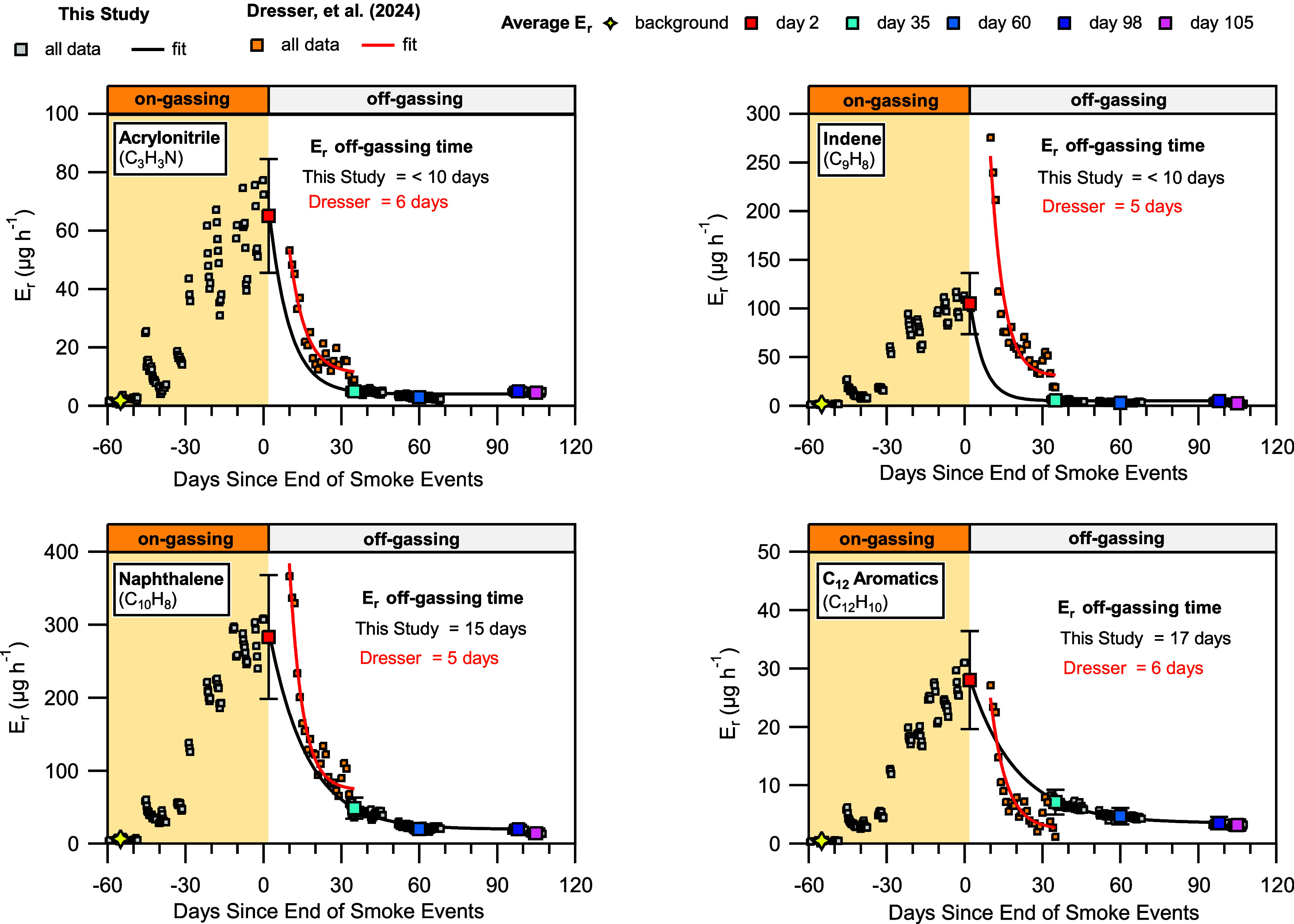
Whole-house emission rates (*E*_r_) of four NMOGs originating from surrogate combustion (gray markers) that were also observed in the house affected by the Marshall Fire from Dresser et al. (orange markers). Lines show first-order decay fits to the data, with black corresponding to the test house and red corresponding to the house from Dresser et al. The orange shaded area shows the period where NMOG surface emission reservoirs were established (on-gassing). The time after smoke events ended (off-gassing) is not colored. Markers (yellow, red, light blue, navy blue, dark blue, and pink) correspond to the average *E*_r_ measured over 5 days. Error bars correspond to the standard deviation (2σ) of that average. The yellow marker corresponds to day −55 (defined as background).

In the 3.5 months following the end of the burn experiments, we observe decreases in NMOG *E*_r_, which we refer to as “off-gassing”. However, because we do not have NMOG measurements at the test house for 1 month immediately after the end of the burn experiments, we are missing data to accurately determine how quickly the *E*_r_ decreased during the off-gassing period. We describe the rate of off-gassing by the *E*_r_ decay time produced from constrained exponential fits (black lines) to *E*_r_ values, which we call the “off-gassing time”. We do not have measurements 2 days after the final burn experiment and thus define a day 2 *E*_r_ (red markers in Figure 2) as the average of the *E*_r_ 2 days prior to the final burn experiment. We constrained the maximum of the exponential decay fit by the day 2 *E*_r_ and the minimum by the average *E*_r_ measured on day 98 (dark blue marker). Measurement data between day 30 and day 98 were included in the fits. We operationally define the off-gassing time as the time for the day 2 *E*_r_ to reach 1/*e* of the difference between day 2 *E*_r_ and day 98 *E*_r_. Because we resumed measurements in the test house 30 days after the end of the burn experiments, we assigned any fits that produce off-gassing times less than or equal to 10 days a value of less than 10 days as the off-gassing time for the NMOG. We estimate day 10 *E*_r_ values from fits of NMOG decays and use those for comparison to day 10 values measured by Dresser et al. in Figure 5 later in the article.

Almost half of the NMOGs (14 of 31) showed off-gassing times of less than 10 days (e.g., acrylonitrile and indene in Figure 2). The minimum off-gassing time we expect a NMOG to have is defined by the λ (λ = 0.2 h^–1^, off-gassing time = 5 h). We conclude that NMOGs like acrylonitrile and indene, whose emissions increase in the test house during the on-gassing period but decrease to background *E*_r_ in less than 10 days, are volatile and weakly partitioned to surface reservoirs. When smoke events occur, these NMOGs are in the gas phase preferentially, but at high concentrations will also partition into surface reservoirs. Our data in Figure 2 suggest that these volatile NMOG contaminants will mostly exit from contaminated buildings due to ventilation on the time scale of a week. Buildings with higher λ values than those used in this study (λ > 0.2 h^–1^) may show a faster decrease in emissions from volatile NMOGs.

As demonstrated by naphthalene and C_12_ aromatics in Figure 2, some NMOGs (10 out of 31) had quantifiable off-gassing times greater than 10 days, with some up to 25 days. NMOGs that showed off-gassing times greater than 10 days typically had *E*_r_ that remained at a value higher than their background through day 98. We also observed that some NMOGs (7 out of 31) did not show clear first-order decay in the off-gassing period, and thus, we did not perform fits for those species (Figure S9 shows benzaldehyde as an example), but the *E*_r_ from these NMOGs also remained elevated above background on day 98. We define this set of NMOGs as having an off-gassing time greater than 25 days. The longer off-gassing times of these NMOGs demonstrate that they likely have more persistent surface reservoirs compared to the more volatile NMOGs. Effective removal of these persistent NMOGs likely requires either surface cleaning (if time to diffuse into the material is minimal) or other forms of remediation to decontaminate. Although determining the mechanism for the prolonged persistence of these NMOGs is beyond the scope of this manuscript, we propose that sunlight irradiating contaminated surfaces (e.g., volatilizing benzaldehyde from surfaces), heterogeneous surface temperature distributions, and multiphase chemistry are all possible reasons why NMOG *E*_r_ could not show a clear first-order decay after the end of the burn experiments.^33^

In Figure 2, we also show *E*_r_ calculated from NMOG mixing ratio measurements presented in Dresser et al. (2024) from a house contaminated by smoke during the 2021 Marshall Fire in Superior, Colorado (hereafter referred to as the Colorado house). The location of the Colorado house was immediately downwind from a block of homes that were completely consumed by the fire.

The initial absolute *E*_r_ values for both the test house and the Colorado house fall within the same order of magnitude for the NMOGs shown in Figure 2. We did not expect agreement of these *E*_r_ values because the contamination that occurred in the Colorado house was produced from a real WUI fire that burned continuously for 3 days, whereas the contamination in the test house occurred over a month and a half from combustion of 11 residential building surrogates lasting 45 min each. Thus, we expected a greater extent of contamination (i.e., higher *E*_r_) in the Colorado house compared to that in the test house. We show examples of larger differences in NMOG contamination from the Colorado house later in the manuscript, but the similarities of the *E*_r_ measured in the test house compared to the Colorado house, shown in Figure 2, suggest that the experimental method used in the current study is reasonable for simulating realistic WUI fire smoke contamination.

The exponential fits to the Dresser data show shorter off-gassing time values compared with the test house for most NMOGs. In particular, the off-gassing times of naphthalene and C_12_ aromatics were at least three times smaller in the Colorado house compared to the test house. All of the NMOGs we analyzed from the Dresser data set show off-gassing times that fall within a relatively narrow window from approximately 3 days to 8 days (Table S4). More data in the month immediately after the end of smoke events in the test house would provide a better quantitative comparison because we would have data that are directly comparable in time to the data available from the Dresser study.

Despite the differences in off-gassing time scales observed from the two data sets, comparison of the results indicates that both buildings experienced elevated NMOG concentrations originating from persistent surface emissions resulting from smoke contamination. Discerning the mechanisms for NMOG persistence (e.g., absorption versus adsorption, chemical transformations) is beyond the scope of the current work. However, several factors could influence the time scales of off-gassing including the λ (i.e., averages for the Colorado house λ = 0.13 h^–1^ and the test house λ = 0.19 h^–1^), differences in construction materials,^34^ house volumes and ages (*V* = 2670 m^3^ home-built in 2020 in Dresser and *V* = 324 m^3^ for test house built in 2002), and chemical reactions on surfaces. These differences could result in different absorption or adsorption kinetics, which would in turn affect the partitioning of NMOGs.^21^

In the following sections, we use the average *E*_r_ values shown as markers in Figure 2 (background, day 2, day 35, day 60, day 98, and day 105) to highlight specific points in time that relate to off-gassing time scales for the NMOGs discussed here. Example *E*_r_ comparisons for select NMOGs are shown in Figure S10.

### Contamination Emission Source Strength

We define the contamination emission source strength in the test house as the background subtracted increase in NMOG *E*_r_ (day 2 Δ*E*_r_ = *E*_r,day2_ – *E*_r,background_) on day 2 after the end of the burn experiments. We find that the day 2 Δ*E*_r_ in the test house is proportional (within a factor of 3) to the corresponding yield of the NMOG, which we previously measured from the surrogates under a hood calorimeter (Figure 3). Figure 3 demonstrates that for our experiments, an NMOG yield of 10 mg of NMOG per kg of combusted surrogate mass creates approximately a 10 μg h^–1^ increase in the day 2 Δ*E*_r_. Because we can approximately predict the emission strength of NMOG contamination as a function of NMOG yield from combustion of the surrogates, we conclude that there are limited relative losses (for the NMOGs evaluated here) impacting the transfer of the smoke from the source outside to the indoors. Thus, the relative composition of the smoke is not changing appreciably between successive burn experiments and the day 2 emissions. This indicates the smoke introduction method used in the test house experiments is reasonable. Additionally, this may indicate that in buildings exposed to real WUI fire smoke, the entry of gaseous contaminants could be approximated if the yields of contaminants from the combustion and the relative amounts of the corresponding materials are known.

**Figure 3:**
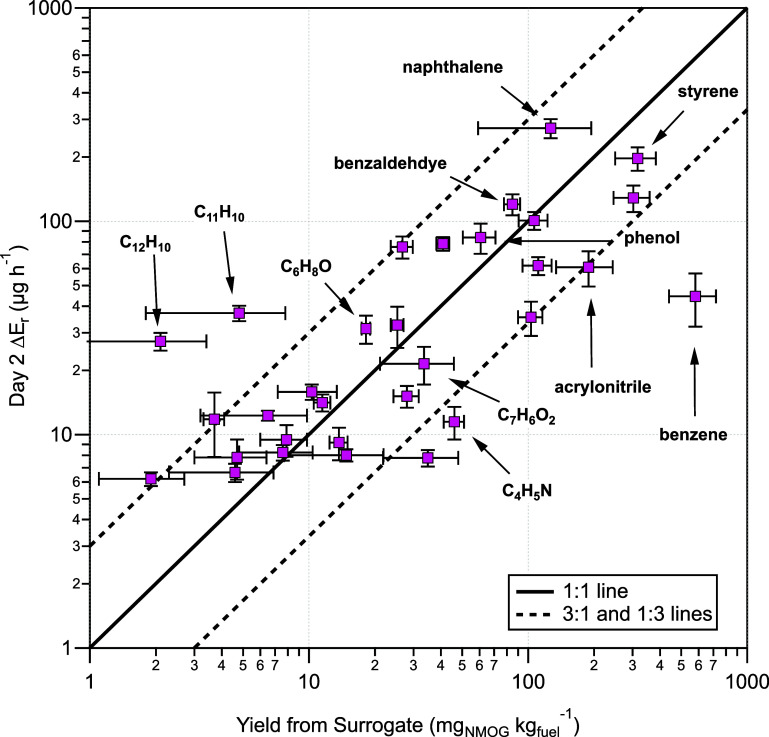
Day 2 Δ*E*_r_ versus the corresponding NMOG yield measured from burning the surrogates under a hood calorimeter reported in a previous study.^23^ One-to-one (solid) and three-to-one (dashed) lines are shown as bounds to interpret data variability. Error bars in the background subtracted *E*_r_ represent the standard deviation (2σ) of the post- and prebackground *E*_r_ added in quadrature. The error bars for the yields show the standard deviation (2σ) of the yields averaged from three surrogate burns under the hood.

### Time Scales of Off-Gassing and Return to Background

The *E*_r_ off-gassing times highlighted in Figure 2 describe a time scale of NMOG off-gassing from the day 2 *E*_r_ value to some *E*_r_ that may be elevated above what we measured for background. We hypothesize that the *E*_r_ off-gassing time may reflect off-gassing from a reservoir like a surface film that may be temporary (i.e., lasting days to weeks). In contrast, if NMOGs show elevated *E*_r_ values above background months after the end of the smoke events, they may be slowly emitting from materials after diffusing into them during the on-gassing period. In Figure 4, we show two quantities associated with NMOG off-gassing time scales to evaluate the persistence of NMOG contamination emission sources on day 35 and day 98: (1) the fraction of day 2 Δ*E*_r_ remaining on days 35 (Figure 4A) and 98 (Figure 4C) and (2) the increase in *E*_r_, compared to background, from NMOG contamination on days 35 (Figure 4B) and 98 (Figure 4D).

**Figure 4:**
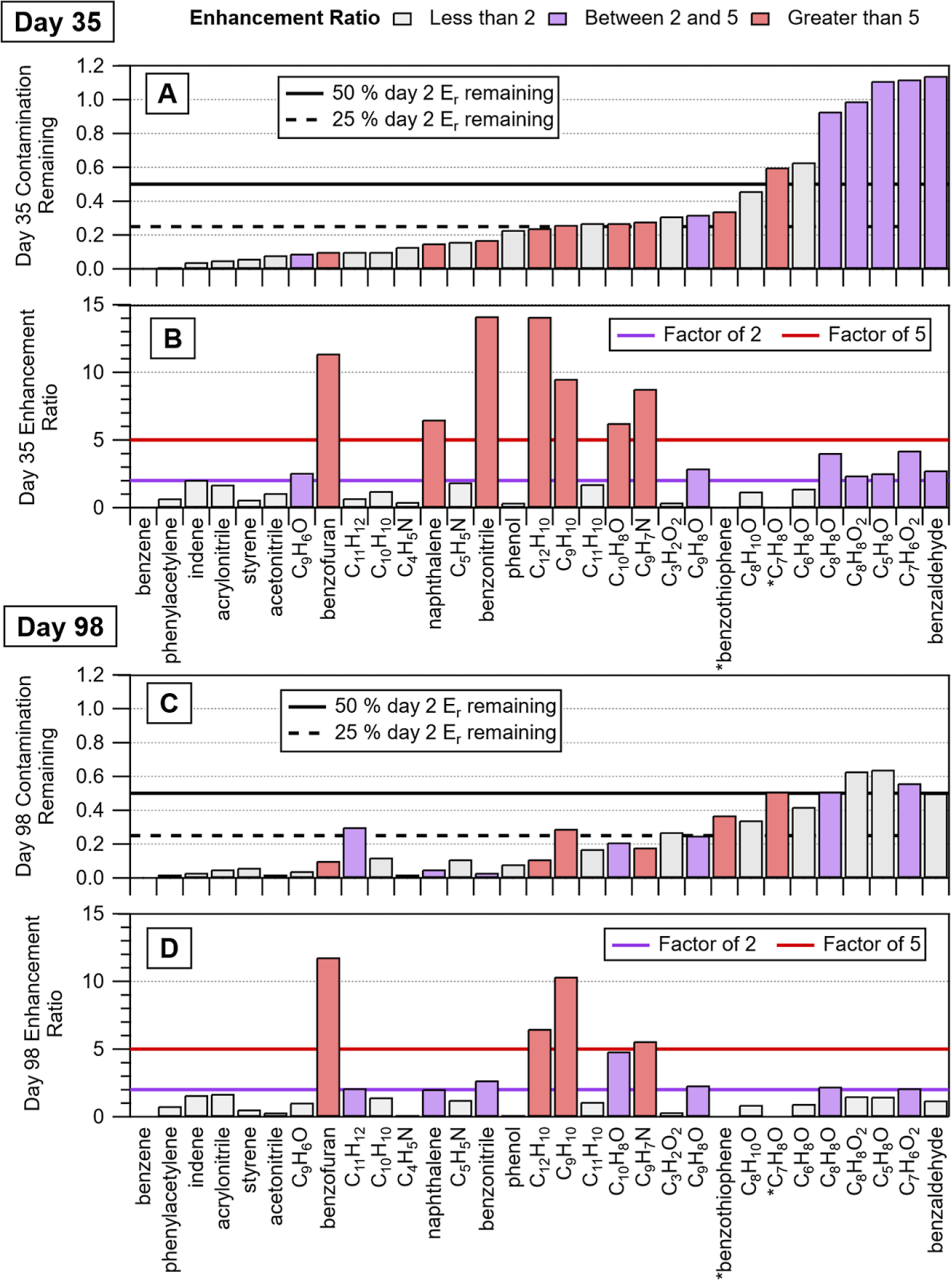
Values measured on day 35 are on the top half and day 98 on the bottom half. Each day contains two panels showing the fraction of day 2 Δ*E*_r_ remaining (panels A and C) and the enhancement ratio (panels B and D). In panels A and C, the solid and dashed lines show the 50 and 25% day 2 Δ*E*_r_ remaining values, respectively. The red and purple lines in panels B and D correspond to enhancements of a factor of 5 and a factor of 2, respectively. Bars are colored similarly in panels A and B and similarly in panels C and D. The colors correspond to enhancement ratios less than 2 (gray), between 2 and 5 (purple), and greater than 5 (red). Benzothiophene and C_7_H_8_O (indicated by an asterisk) were not detected in the test house background, and thus, we cannot calculate enhancement ratios for those NMOGs.

We define the fraction of remaining NMOG contamination after day 2 (*F*_day35or98_) following eq 2

2 $$F_{day35\text{or}98}=\frac{\Delta E_{r,day35or98}}{\Delta E_{r,day2}}$$

where Δ*E*_r_,_day35or98_ is the background-subtracted *E*_r_ for day 35 or 98 and Δ*E*_r_,_day2_ is the day 2 Δ*E*_r_. If none of the NMOG contamination initially introduced during the on-gassing period (i.e., day 2 Δ*E*_r_) is present on day 35 or day 98, then we would expect the day 35 or day 98 background-subtracted *E*_r_ to be close to zero and thus the fractions in Figure 4A,C to also be close to zero. The purpose of this analysis is to understand how long (if at all) it will take for NMOG contamination to off-gas completely, given enough time, without any remediation.

The fraction of contamination remaining is relative to the absolute increase in day 2 *E*_r_ incurred during the on-gassing period. Thus, in addition to quantifying how much NMOG contamination remains during the off-gassing period, we quantify how much the remaining emissions are elevated compared to the background. We define an NMOG enhancement ratio following eq 3

3 $$ER_{day35\text{or}98}=\frac{E_{r,day35\text{or}98}}{E_{r,background}}$$

where *E*_r,background_ is the background *E*_r_ (day −55 *E*_r_) and the ER_day35or98_ is the enhancement ratio for a NMOG measured on day 35 or 98. Enhancement ratios equal to one mean that day 35 or 98 *E*_r_ values are equal to the background. We show lines in Figure 4B,D corresponding to enhancement ratios of two and five to describe relative enhancements.

By day 35, much of the NMOG contamination quantified in this study exited the building through ventilation because approximately half of the NMOGs (16 NMOGs) showed *E*_r_ less than 25% of their day 2 Δ*E*_r_ (Figure 4A). However, seven of those NMOGs (benzofuran, naphthalene, benzonitrile, C_12_ aromatics, C_9_H_10,_ C_10_H_8_O, and C_9_H_7_N) showed enhancement ratios that were at least a factor of 5 higher than background (Figure 4B). Although these seven NMOGs had off-gassed to 75% of their maximum emissions measured on day 2 by day 35, their emissions remained considerably elevated above background levels. In contrast, seven NMOGs showed remaining day 2 fractions higher than 50%, but also had enhancement ratios close to two. The day 2 emissions from contamination were persistent for these NMOGs, but their absolute emissions and effects on indoor air concentrations were relatively small (about a factor of 2 higher than background).

By day 98, the contamination emission source strength of nearly all of the NMOGs highlighted in Figure 4C (except three) had decreased to at least 50% of their day 2 Δ*E*_r,_ with most (20 NMOGs) showing a decrease of at least 75%. For several of the NMOGs where the fraction of day 2 Δ*E*_r_ remaining is close to 50% (benzaldehyde, C_7_H_6_O_2_, C_5_H_8_O, C_8_H_8_O_2_, and C_8_H_8_O), the corresponding enhancement ratios are close to two (Figure 4D), indicating that, although they may exhibit persistent emissions, their contamination emission source strengths on day 2 (i.e., day 2 Δ*E*_r_) were no more than a factor of 4 higher than background. In contrast, several NMOGs (benzofuran, C_12_H_10_, C_9_H_10_, and C_9_H_7_N) that show fraction remaining values no greater than 25% also show enhancement ratios greater than 5. The day 2 Δ*E*_r_ of these NMOGs was a factor of 20 or greater than background and continued to show high emissions (compared to background) three months after the end of the smoke events.

Finally, many NMOGs on day 35 (expanding to a larger set on day 98) showed fractions remaining less than 25% and enhancement ratios less than two. Some of these NMOGs, like acrylonitrile, styrene, and indene, we have previously identified as volatile (meaning that they did not have high *E*_r_ off-gassing times) and did not show elevated emissions in the months after the smoke events. For these volatile NMOGs, although the day 2 Δ*E*_r_ may have been high (compared to background), most of the contamination was ventilated from the test house by day 98, and emissions were not elevated (more than a factor of 2) above background.

Although it is outside the scope of the current work to discern the mechanisms for NMOG persistence, we observe that both fraction remaining values and enhancement ratios do not show a clear relationship to their octanol–air partitioning coefficients (*K*_OA_) or vapor pressures (Figures S11 and S12). K_OA_ is defined as the ratio of the NMOG octanol–water partitioning coefficient to the Henry’s law constant. Larger values indicate a greater partitioning of the NMOG to an octanol-equivalent film (weakly polar surface reservoir) than that of the gas phase at equilibrium. Previous studies of semivolatile gases in indoor air have demonstrated that organic molecules with larger log­(*K*_OA_) (typically greater than 5) will slowly evaporate from surfaces and affect indoor air on longer time scales (days to years), whereas molecules with smaller log­(*K*_OA_) (typically less than 5) will evaporate from surface reservoirs rapidly and thus affect indoor air on shorter time scales (hours to days).^14,21,35,44^ Future modeling could possibly quantify the adsorption kinetics that result in NMOG persistence in the test house.

### Removal of the Carpet

We removed the carpet from the test house approximately 100 days after the end of the burn experiments to evaluate it as a source of NMOG contamination. Approximately 80% of the floor area of the test house was covered in carpet. When comparing the day 98 *E*_r_ (several days before carpet removal) to the day 105 *E*_r_ (several days after carpet removal), carpet removal produced a 20% decrease in NMOG emissions (Figure S13). We also recalculated *E*_r_ values using a constant ACR value (λ = 0.14 h^–1^) and plotted the day 98 values against the day 105 values to understand whether the change in the ACR estimated from the polynomial fit was creating a bias in the relationship. Going from day 98 to day 105, *E*_r_ calculated from a constant ACR value decreased by nearly 40% indicating decreases in mole fractions and not changes in ACR are likely driving the decrease in emissions.

However, our estimates of decreased emissions of smoke contaminants are limited, because we could not account for the decrease in *E*_r_ that may have occurred if we had not removed the carpet. Additionally, decreases in *E*_r_ may be related to emissions from the carpet material and not necessarily deposited smoke. As shown in Figure 4C, by day 98 (several days before the carpet was removed), most of the NMOG *E*_r_ values were less than 25% of their maximum on day 2, making it difficult to accurately quantify the effect of carpet removal on smoke contamination. However, we note that contaminants (e.g., semivolatile gases or particles) could be deposited on the carpet that we could not measure with the PTR-MS, because they are effectively nonvolatile, and thus removal of the carpet could possibly be an effective way to decontaminate homes and reduce exposure to those types of contaminants. Considering the limitations of our experimental approach, we suggest that a more thorough evaluation of the role of carpet as an NMOG emission source after contamination is warranted.

### Comparison of Emissions Measured in the Test House versus a House Contaminated with Real WUI Fire Smoke

We compare the emission source strength in the test house, from the 11 surrogate burns over 1.5 months, to the Colorado house (measurements from Dresser et al.), to evaluate if our experiments adequately replicate the amount of contamination a building exposed to real WUI smoke may experience. Figure 5 shows a comparison of NMOG *E*_r_ from the Colorado house and test house 10 and 35 days after the end of the smoke events. Measurements 10 days after the end of the smoke events were not available for the test house, and thus, values are estimated from the *E*_r_ decay fits shown in Figure 2.

**Figure 5:**
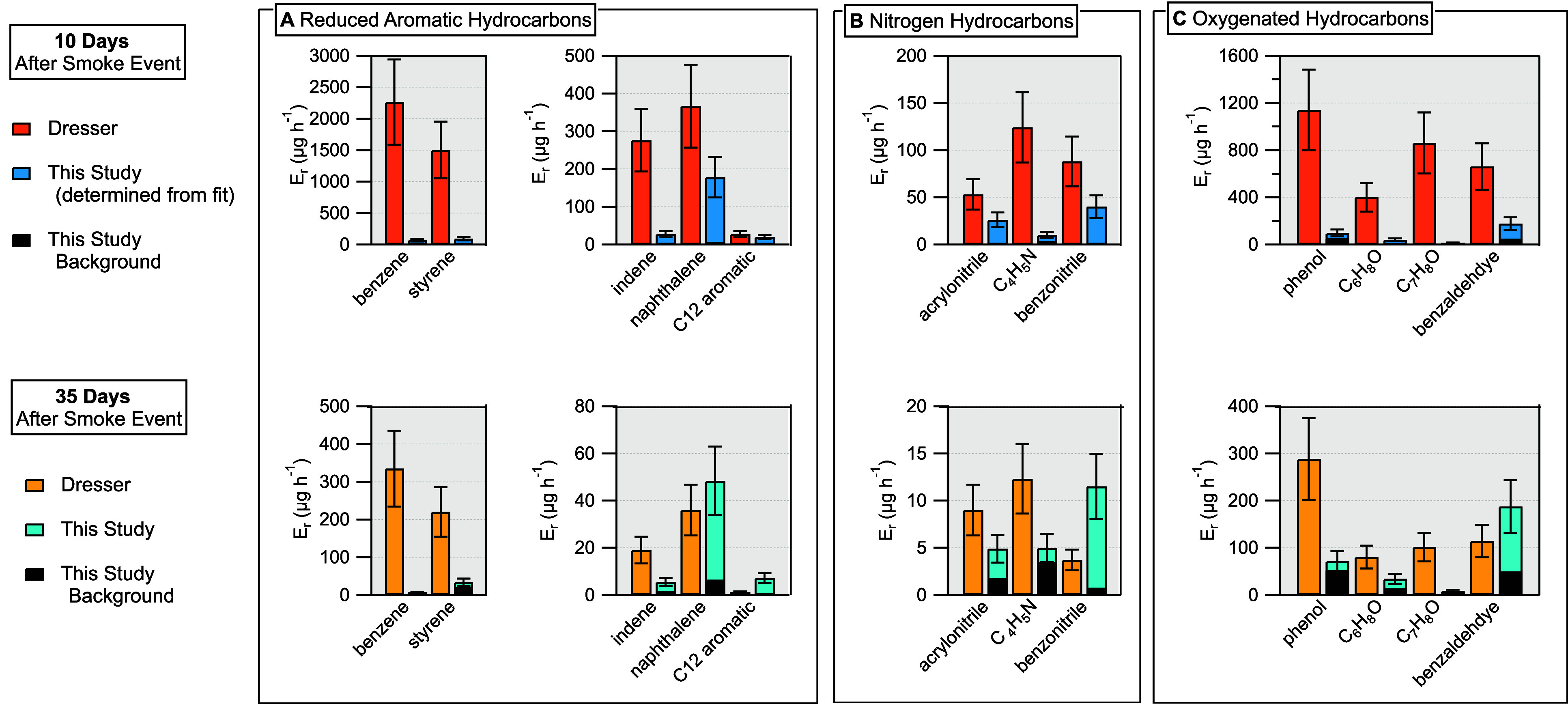
Comparison of *E*_r_ for select NMOGs from different chemical classifications (reduced aromatic hydrocarbons, nitrogen-containing hydrocarbons, and oxygenated hydrocarbons). Top panels show *E*_r_ measured 10 days after the smoke event from the Colorado house, compared to the day 10 *E*_r_ estimated from a first-order decay fit to test house *E*_r_ data from day 2 to day 98. Black bars show the measured background *E*_r_ in the test house. Background contributions are more visible in the bottom panels versus the top panels. Bottom panels show *E*_r_ measured 35 days after the end of the smoke events. Descriptions of NMOGs identified by their molecular formula can be found in Table S1. Error bars for *E*_r_ values from both the Colorado house and test house show the 30% uncertainty originating from the uncertainty in the respective measurements of the λ.

Nearly all NMOGs highlighted in Figure 5 showed higher *E*_r_ from the Colorado house on both days 10 and 35 compared to the test house, except for benzonitrile, naphthalene, and benzaldehyde on day 35.

The *E*_r_ values for benzene, styrene, and indene were much higher in the Colorado house for both 10 days (100× higher for benzene and styrene) and 35 days after the end of the smoke events (Figure 5A). Naphthalene and C_12_ aromatics had emissions that fell within the uncertainty for both houses. The discrepancy between the high emission values of benzene and styrene observed from the Colorado house compared to our study suggests the real WUI smoke may have been more enriched with those NMOGs compared to the surrogate smoke. However, we note that, using gas chromatography preseparation, Zhang et al. (2025) recently quantified major ion interferences with the proton-transfer product ions for benzene (C_6_H_7_^+^) and styrene (C_8_H_9_^+^) in urban air, showing that contributions of signals from product ions of other NMOGs could create positive interferences. Because of possible contributions from ion interferences, the *E*_r_ values of benzene and styrene calculated from data from the Dresser study may represent upper limits.

The *E*_r_ for acrylonitrile and benzonitrile are nearly equivalent in the day 10 comparison, whereas the *E*_r_ for C_4_H_5_N compounds is higher in the Colorado house. C_4_H_5_N measured from the test house is a sum formula comprised of pyrrole and C_4_ nitriles that have been reported from combustion of biomass,^37^ and the higher emissions from the Colorado house may indicate a greater influence of biomass combustion (both structural and otherwise) compared to the surrogate smoke.

Similarly, for all of the oxygenated hydrocarbons in Figure 5C, emissions are higher on both days 10 and 35 in the Colorado house compared to those in the test house (except for benzaldehyde on day 35). We recently reported lower yields of oxygenated hydrocarbons from the surrogates (measured under a hood calorimeter) compared to what has been reported for biomass,^23^ and the higher emissions of oxygenated hydrocarbons in the Colorado house may indicate a more pronounced influence of biomass-like smoke contamination compared to the test house.

The black “background” bars superimposed on the measurements from the test house in Figure 5 demonstrate that background emissions made important contributions to the total *E*_r_ values measured 35 days after the end of smoke events. Although the Dresser study did not quantify background NMOG emissions from the Colorado house, the comparison shown in Figure 5 between background and nonbackground emissions in the test house suggests that the emissions shown from the Colorado house are likely upper limits of emissions from smoke contamination.

There are many possible reasons why differences in emissions measured from the test house compared to the house in Colorado exist, but we want to highlight several important observations from this comparison: (1) more than a third of the NMOGs (12 out of 31) quantified as having persistent emissions in the test house were also measured as smoke-derived contaminants in the Colorado house, (2) NMOG emissions appear to undergo first-order decay from both houses, and (3) after 35 days, most of the NMOGs measured from both houses showed emissions that were within a factor of 2 to 3 of one another. Together, these observations lead us to conclude that changes in smoke composition from different surrogate designs and a greater understanding of the mechanisms controlling NMOG partitioning from surfaces to indoor air could help us improve our ability to replicate real WUI fire contamination in future studies.

## Implications

The most volatile NMOGs highlighted in this study (e.g., acetonitrile, acrylonitrile, styrene, indene, etc.) off-gassed from both the test house and the Colorado house at rates faster than other NMOGs, but much slower than predicted by ventilation alone. Thin films on indoor surfaces have been shown to regulate adsorption and sorption kinetics of NMOGs, with thicker films creating more persistent emission sources than thinner films.^21,44^ However, given sufficient loading, NMOGs can diffuse into materials and also create persistent emission sources. The relatively fast off-gassing (i.e., low *E*_r_ off-gassing times) of many volatile NMOGs highlighted here likely points to a thick surface film that creates a relatively strong, but transient emission source. However, persistent emissions enhanced above the background for many NMOGs over a period of months in the test house may also point to an emission source from gases embedded within materials and/or strongly partitioned to surface films. Surface cleaning may be an adequate remediation method for surface film reservoirs^19^ but may not be adequate for contaminant gases embedded into materials.^38^ Ventilating a contaminated home as much as possible for at least several days after a smoke event could promote NMOG removal faster than we observed.

In this study, we did not evaluate the persistence of contaminants of concern that are not selectively measured with PTR-MS. For instance, Wang et al. (2025) recently found that hydrochloric acid (HCl) was among the most abundant toxicant gas emissions produced from combustion of urban materials relevant to WUI fires. The PVC in our surrogates likely produced a high yield of HCl (as measured from hood calorimeter experiments), but we did not have instrumentation to measure HCl from the test house. HCl is expected to undergo rapid deposition,^35,39^ and then re-emit from surface reservoirs,^40,41^ thus likely persisting in indoor air on the week-long time scales observed for NMOGs from the test house. Additionally, we did not evaluate the persistence of particle-bound contaminants, such as polycyclic aromatic hydrocarbons and metals. These nonvolatile contaminants will not be removed by ventilation at all and can remain indoors on surfaces or be scavenged by dust even after surface cleaning.^38^

Through comparison of NMOG *E*_r_ measured in the test house from simulated WUI fire smoke contamination to *E*_r_ measured from a house contaminated with smoke from the Marshall fire, we demonstrated our experimental method produces levels of contamination that are reasonable but likely less than what can occur from real WUI fires. Importantly, real WUI fires involve complex combustion processes dependent on fuel type, fuel load, and combustion mechanisms (e.g., flaming versus smoldering combustion) that likely were not captured by contaminating the test house from our surrogates. For instance, the higher emission rates of naphthalene, benzonitrile, and benzaldehyde in the test house compared to the Colorado house 35 days after the end of the smoke events are likely associated with the emissions from the synthetic polymers and mixed fuel char of the surrogates,^23^ and the real WUI smoke may not have been as concentrated with these NMOGs.

Future work from our group using this experimental design will focus on assessing the effectiveness of remediation techniques and quantifying contamination by gases and particulate matter not highlighted in this study. Many of the NMOGs presented in this work come from other sources that can contaminate indoor air, including wildfires^9,16,22^ and industrial accidents,^42,43^ and thus, our results may have practical implications for assessing contamination from those sources. Real-time measurements in the days and weeks immediately following the on-gassing period could provide data that would be helpful in assessing the NMOG partitioning capacity and enable a more informative comparison between our experiments and real-world measurements.

## Disclaimer

Certain equipment, instruments, software, or materials are identified in this work in order to specify the experimental procedure adequately. Such identification is not intended to imply recommendation or endorsement of any product or service by NIST, nor is it intended to imply that the materials or equipment identified is necessarily the best available for the purpose.

## Supplementary Material



## Notes

Simplified schematic of the NIST test house, detailed information on the parametrization of the air change rate and supplemental sensitivity analysis, tabulated details on NMOG identification, chemical properties, and average emission rates, and figures showing supplementary analyses of emission rates with chemical properties (PDF)

## $

University of Colorado Boulder, Boulder, 80309, Colorado, United States
