# Supplementary material for: Time Scales of Gaseous Smoke Contamination Indoors from Real and Simulated Wildland-Urban Interface Fires
Source: ACS EST Air. 2025 Dec 5;3(1):224–35. doi: 10.1021/acsestair.5c00358 (PMC12797220; doi:10.1021/acsestair.5c00358)
Supplement: Supplementary file 1 [file ea5c00358_si_001.pdf]

Supplemental Information for:

Timescales of Gaseous Smoke Contamination Indoors from Real and Simulated Wildland-Urban Interface (WUI) Fires

Michael F. Link, Aika Y. Davis, Nathan M. Lima, Ryan L. Falkenstein-Smith, Rileigh L. Robertson*[$], *Thomas G. Cleary, Steven Emmerich, Dustin Poppendieck*

National Institute of Standards and Technology, Gaithersburg 20899 Maryland, United States

[$]Now at: University of Colorado Boulder, Boulder 80309 Colorado, United States

[*]*Corresponding author email: michael.f.link@nist.gov*

Number of Pages: 22

Number of Figures: 13

Number of Tables: 4

Simplified Diagram of the NIST Manufactured House ("test house").

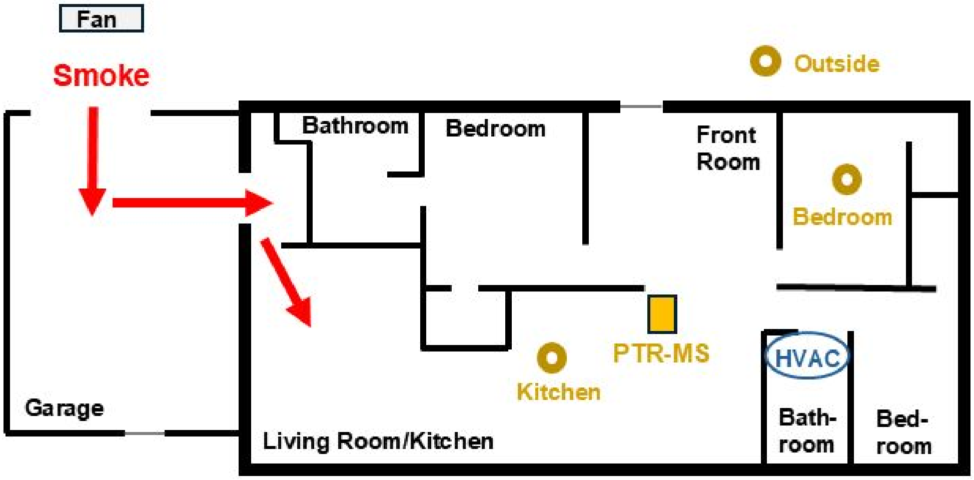

Figure 1. Simplified diagram of test house. Smoke from the surrogate enters the indoors via a door between the garage and living room/kitchen (red arrows). The proton-transfer reaction mass spectrometer (PTR-MS) was located inside and alternately sampled from three locations (outside, kitchen, and bedroom). SF_6 was injected into the HVAC intake (location indicated in blue) and distributed throughout the test house.

Measurements of Air Change Rate (λ) using SF_6 and Methane (CH_4). As described in the main text, SF_6 injections were performed frequently during the measurement period between April and mid-September to calculate the outdoor air change rate (λ) through tracer decay analysis. Complimentary to our determinations of the λ using SF_6, we also used indoor and outdoor measurements of CH_4 combined with a mass-balance calculation to determine CH_4-dervied λ values. We used the λ values determined with the CH_4 method to assess if fitting a polynomial to λ values derived with SF_6 data was appropriate when there were multi-day gaps in λ data in the spring. The λ values used to calculate NMOG emission rates in the main text come from the polynomial fit to the SF_6 λ values.

To calculate CH_4-dervied λ we ignored data 48 hours immediately after a burn experiment, then rearranged Equation 1 from the main text to solve for the λ term. We assumed a quiescent

emission rate of 0.33 g day^{-1} with a relative standard deviation of 26 % as measured over the previous two seasons from the test house.[1] Figure S2 shows a comparison of SF_6 (black markers) and CH_4 (blue markers) derived λ values. Because of interruptions in the SF_6 injection and measurement system we fit the λ (derived from SF_6 measurements) to a polynomial function to estimate the λ during periods without data. We apply an uncertainty of 30 % to the fit as that uncertainty encompasses most available data. The parameterized λ was not applied to the brief period between April 5 and April 8 where the λ was approximately a factor of two higher than the rest of the measurement period. The λ was elevated during this period because an exhaust fan was running in the test house. For these three days we used the average of the λ (measured from CH_4) of 0.43 h^{-1}.

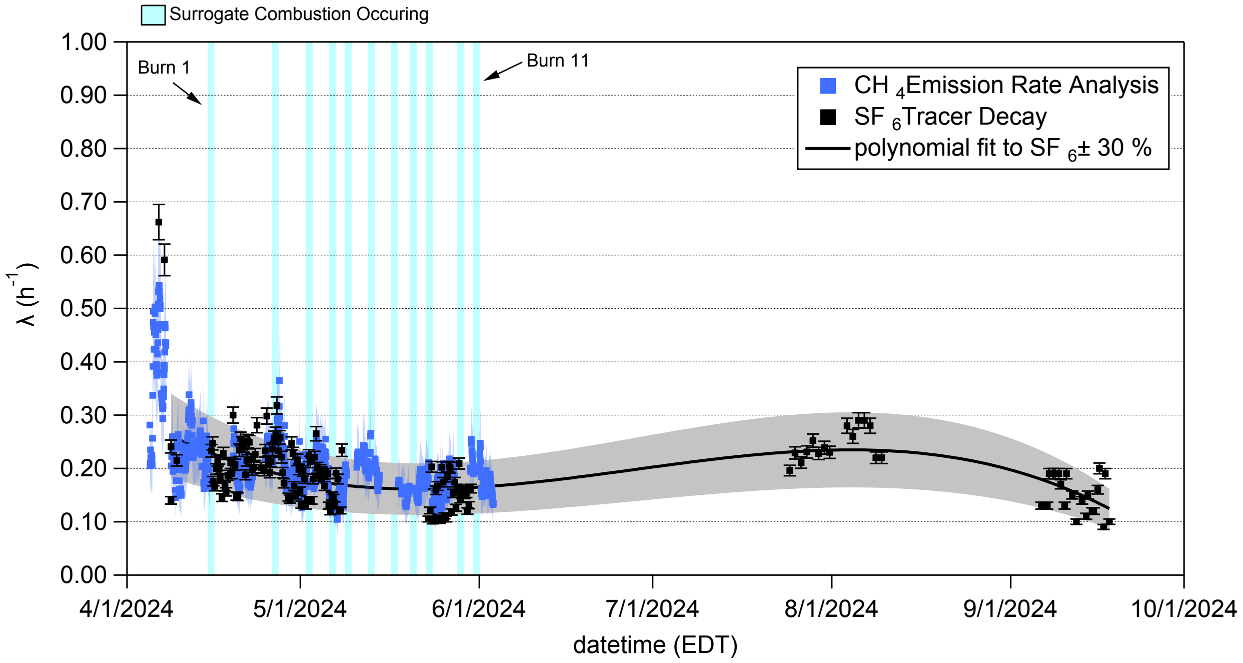

Figure S2. λ values determined from SF_6 tracer decay analysis (black markers) and from CH_4 emission rate analysis (blue markers). SF_6 λ values were fit with a four-parameter polynomial with an estimated uncertainty of 30 %. Blue shaded areas shown the approximate time surrogates were burned outside the test house.

A fourth order polynomial was chosen for the parameterization of λ because more variability in the measured λ was captured going from third to fourth order, but no improved agreement between measured and fitted data was observed when going from fourth to fifth order (Figure S3). Figure S3 also shows that the fourth order polynomial captures more of the time-dependent variability in λ data than the campaign average.

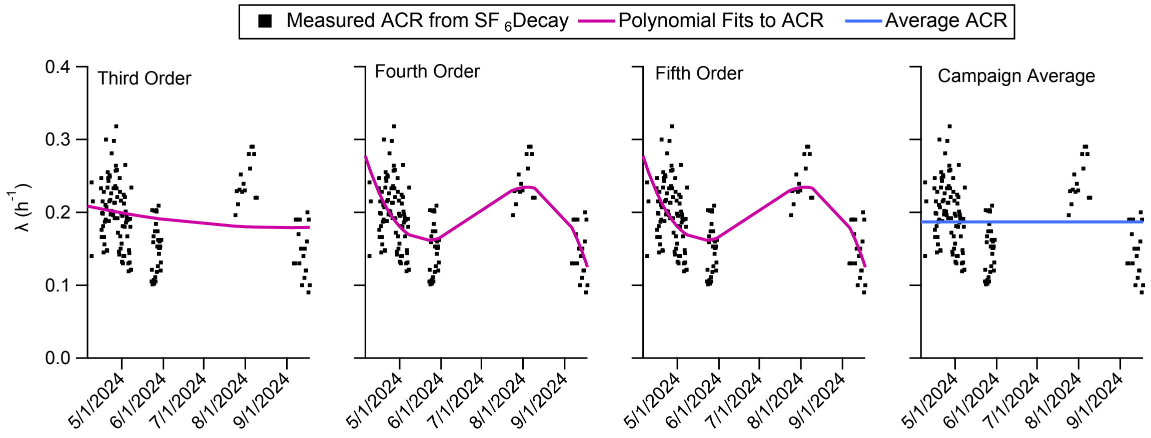

Figure S3. Comparison of polynomial fits of λ values to the campaign average λ of 0.19 h^{-1}.

We acknowledge that the choice of using a polynomial to fit the available λ data versus time is not founded in a physical basis. Thus, we compare the E_r calculated for acrylonitrile and indene using λ derived from the polynomial parameterization to the campaign average λ in Figure S4. We find that E_r calculated using λ from the polynomial fit may be approximately 13 % lower compared to E_r calculated using the campaign-average λ.

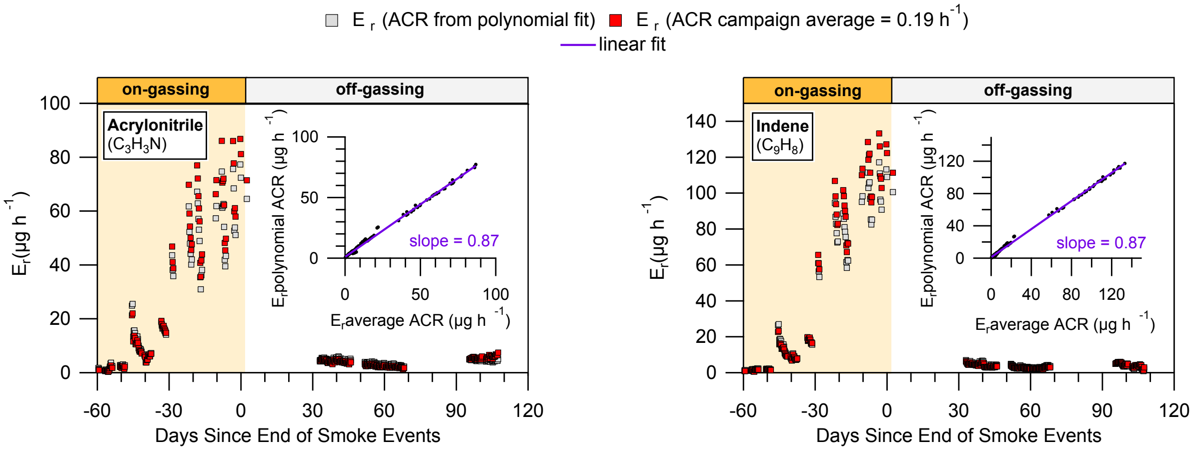

Figure S4. E_r for acrylonitrile and indene calculated using λ values derived from the fourth-order polynomial fit (gray markers) compared to the campaign-averaged λ (red markers). Inset panels showing a linear correlation between gray marker values and red marker values.

Figure S5 shows the direct comparison of CH_4 to SF_6 derived λ values measured directly at the same time. The one-to-one line is also shown, and the data are bounded by ± 50 % on the one-to-one line. Although most measurements fall within ± 50 % of the one-to-one line, the average ratio of SF_6 values to CH_4 values was calculated to be 1.01 ± 0.26.

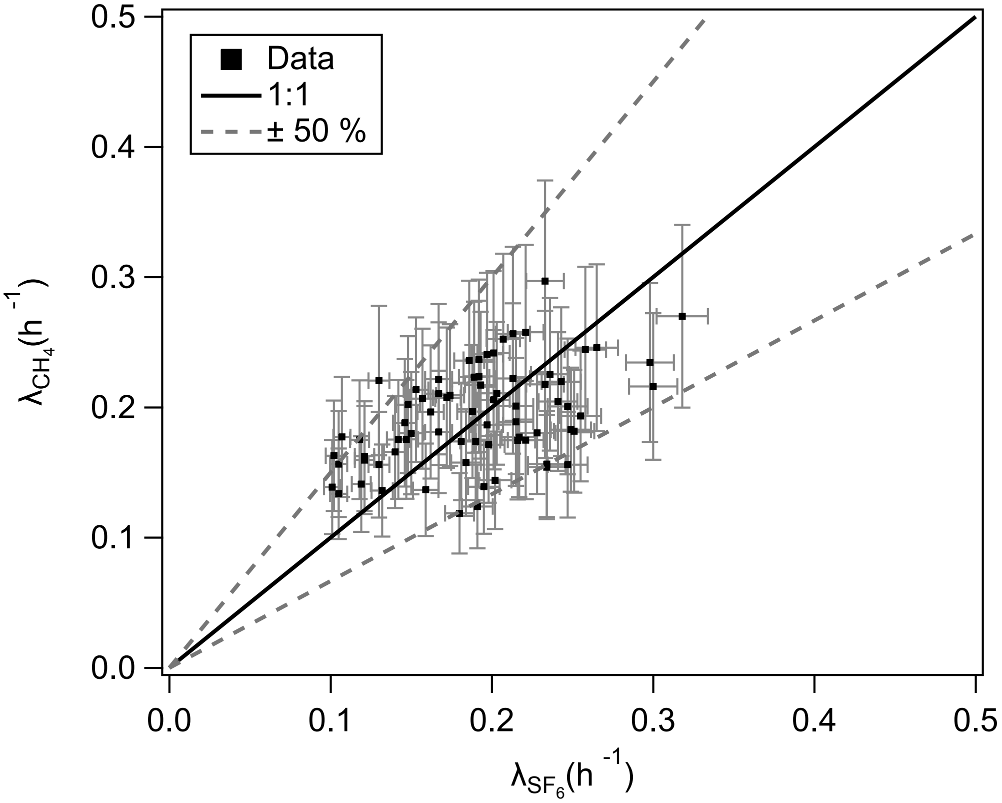

Figure S5. Direct comparison of λ values measured coincidently from both CH_4 emission and SF_6 decay analyses. The solid black line shows the one-to-one relationship, and the dotted lines show the ± 50 % from the one-to-one. Vertical error bars are the ± 26 % uncertainty on the CH_4-dervied λ and the horizontal error bars show the ± 5 % uncertainty in SF_6-derived λ values propagated from the uncertainty in the tracer decay linear fit analysis.

Characterization of PTR-MS ion signals, quantification of NMOGs, and summary of E_r.

We filtered ions measured by the PTR-MS to differentiate NMOGs originating from WUI surrogate smoke contamination and those originating from background emissions in the test house using the following criteria:

1. Only analysed ions previously measured from surrogate emissions under a hood calorimeter (201 ions).

2. After converting ion signals to mole fractions we only quantified emission persistence from NMOGs whose background mole fractions increased incrementally with each successive burn during the on-gassing period.
3. From this set of NMOGs we identified several species that had problematic product ion interferences and removed them. For example, the $C_7H_9O_2^+$ ion can be identified as the the proton-transfer product for guaiacol, but was determined to be a water cluster of benzaldehyde in our experiments through analysis with a PTR-MS coupled to a gas-chromatograph (PTR-MS-GC).

This filtering analysis left us with 31 NMOGs that we report emission rates for in the main text. Of the 31 NMOGs, several of the ion signals associated with the NMOGs were comprised of multiple isomers and/or had partial signal contributions from product ion interferences. Table S1 shows the 31 NMOGs we focus our analyses on in our study. We report the number of peaks identified in the chromatograms (measured with GC-PTR-MS) acquired in August 2023 (nine months before the beginning of the bur experiments representing test house background), before one of the last burns (showing NMOG contributions approximately 2 days after a surrogate burn), and an hour after one of the last burns (showing major NMOGs originating from surrogate smoke). Example chromatograms from these different times are shown in Figures S6 through S8.

Based on the number of peaks in the GC chromatograms and the strength of the signals we follow a similar method recently reported in Zhang, et al. (2025) and assigned a confidence category to the ion for which we calculated NMOG E_r.

- <u>Category I:</u> Ions that have only one peak in the chromatogram and are clearly identifiable from retention time. The uncertainty associated with mole fractions calculated from these compounds is clearly defined by the calibration uncertainty (15 % for direct calibrations and 50 % for indirect calibrations).

- Category II: Ions that have multiple peaks in the chromatogram attributable to isomers and are minimally affected by product ion. The uncertainty associated with mole fractions calculated from these compounds is greater than that of category I compounds. However, the source of the uncertainty is mostly attributable to possible differences in sensitivity between isomers.
- Category III: Ions that have multiple peaks in the chromatogram with contributions from product ion interferences and/or did not elute in GC analysis. The uncertainty associated with mole fractions calculated from these compounds is difficult to quantify. Often with these ions it is difficult to determine what is the signal from a real NMOG and what is a product ion interference (water cluster, fragment, etc.).

Figures S6 though S8 show examples of category I, II, and III compounds respectively. In those figures we compare test house background (top panels, before any smoke contamination), test house contaminated background (middle panels, 48 hours after a burn and the evening prior to the penultimate burn), and the test house two hours after a burn experiment (bottom panels). Background GC measurements were acquired in August 2023, prior to a single preliminary burn in the fall of 2023.

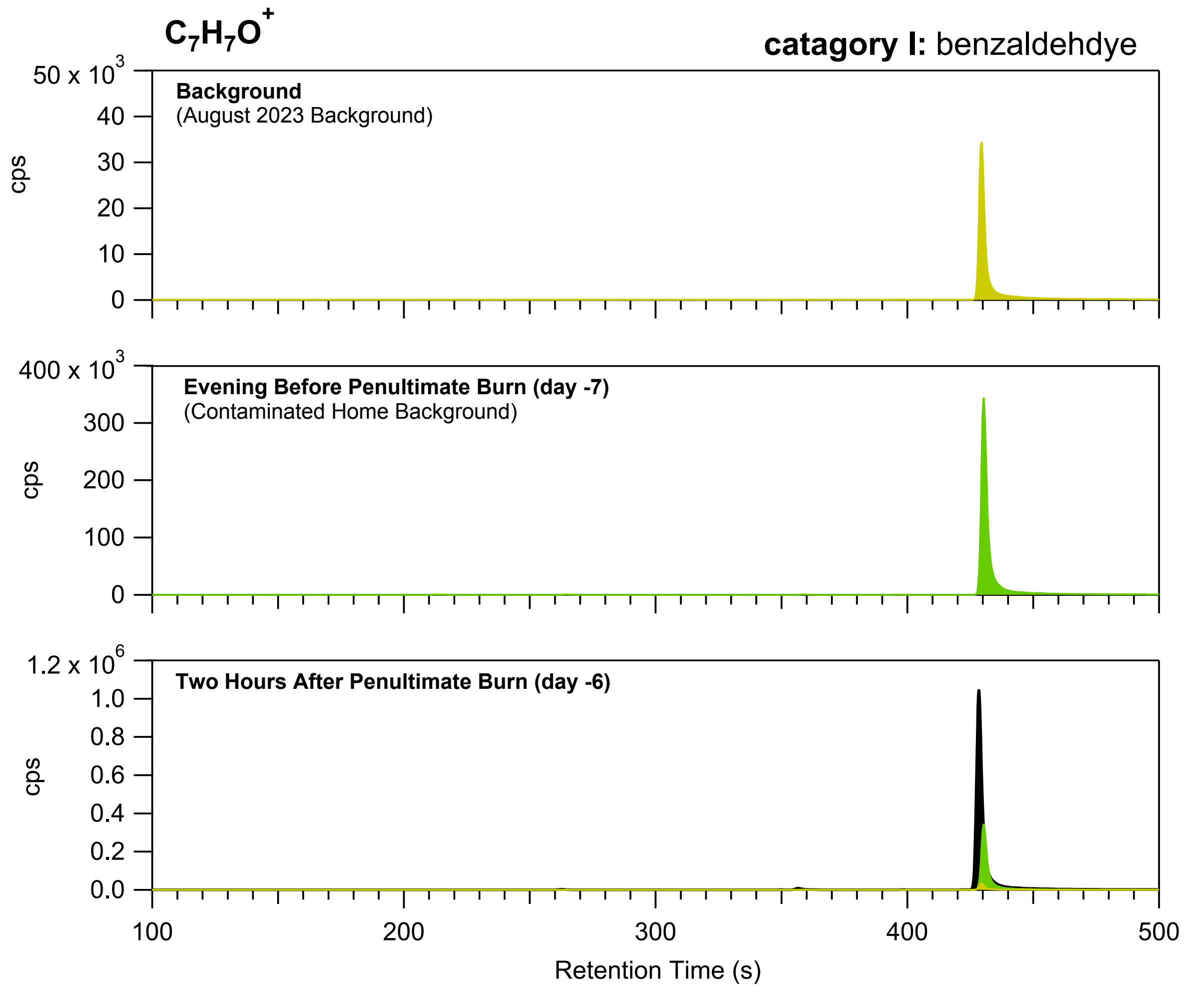

Figure S6. Selected ion chromatograms for $C_7H_7O^+$ which is identified as a category I NMOG. The chromatograms for background and day -7 are superimposed on the day -6 chromatogram to demonstrate the higher magnitude in signal for $C_7H_7O^+$ immediately after a burn experiment.

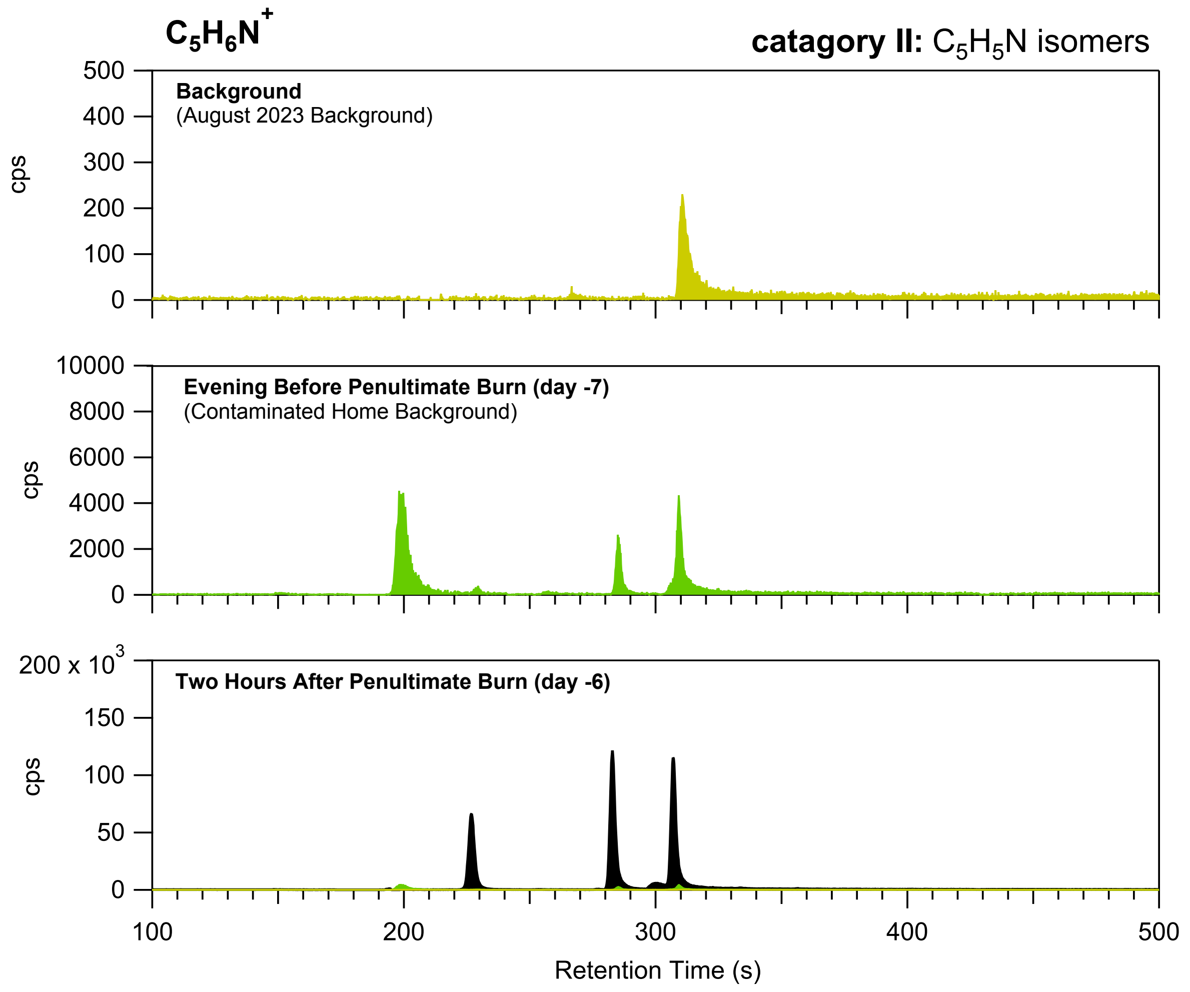

Figure S7. Selected ion chromatograms for $C_5H_6N^+$ which is identified as a category II NMOG. The chromatograms for background and day -7 are superimposed on the day -6 chromatogram to demonstrate the higher magnitude in signal for $C_5H_6N^+$ immediately after a burn experiment. The peaks below 300 s retention time are isomers. All peaks correspond to NMOGs with a molecular formula of C_5H_5N.

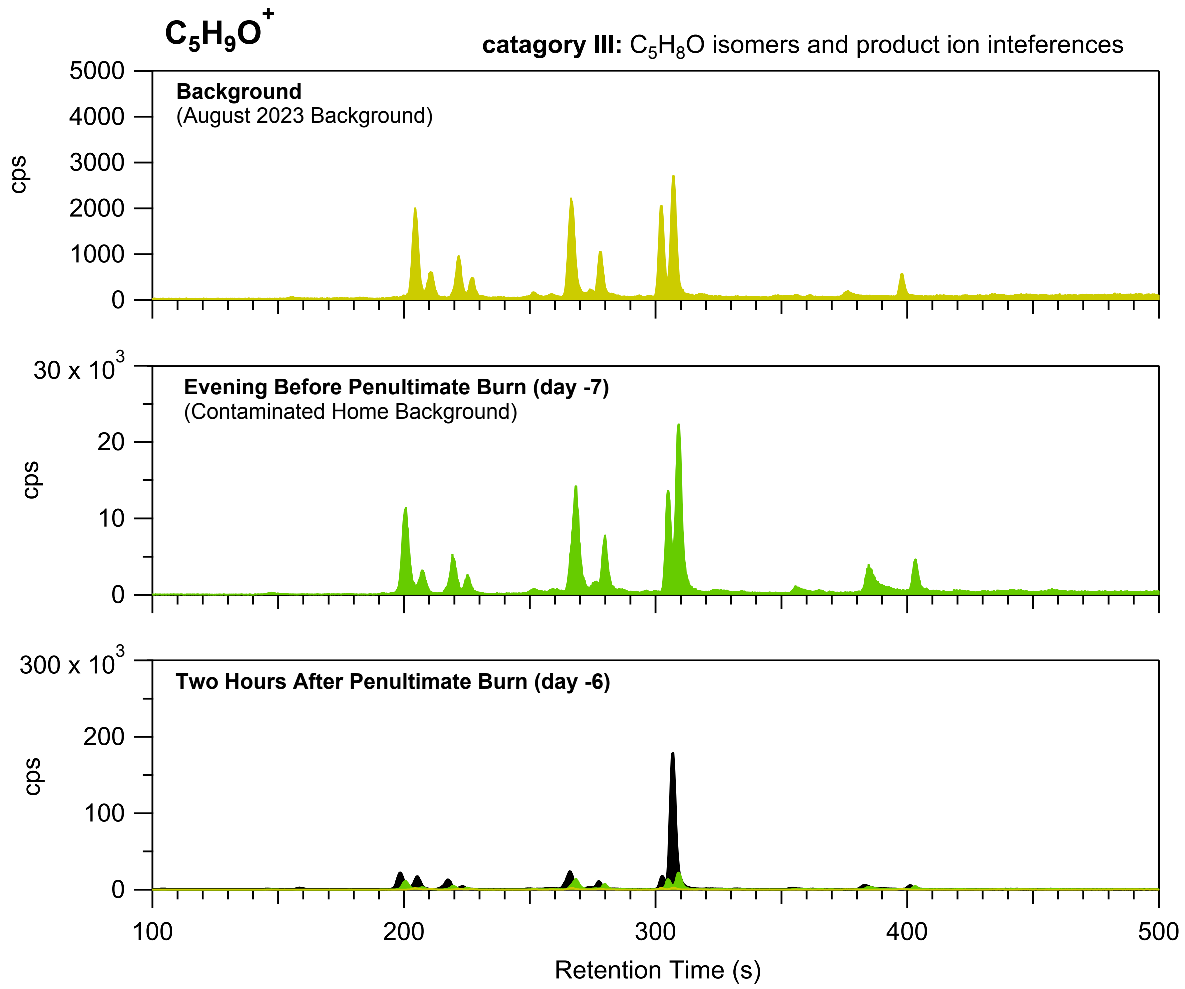

Figure S8. Selected ion chromatograms for $C_5H_9O^+$ which is identified as a category III NMOG. The chromatograms for background and day -7 are superimposed on the day -6 chromatogram to demonstrate the higher magnitude in signal for $C_5H_9O^+$ immediately after a burn experiment.

Table S1. PTR-MS NMOG measurement details.

			Number of peaks in GC chromatogram				
Ion formula*	m/Q	ID**	Back-ground	Pre-burn	Post-burn	Confidence Category	Comment
$C_2H_4N^+$	42.03	acetonitrile	1	1	1	I	
$C_3H_4N^+$	54.03	acrylonitrile	1	1	1	I	

$C_4H_6N^+$	68.05	pyrrole and C_4 nitriles	5	5	5	II	
$C_3H_3O_2^+$	71.01	propiolic acid and product ion interferences	2	9	10	III	
$C_6H_6^+$	78.05	benzene and product ion interferences	5	5	2	III	Benzene is > 50 % of signal for all chromatograms
$C_5H_6N^+$	80.05	pyridine and cyanobutadiene isomers	1	3	3	II	
$C_5H_9O^+$	85.06	C_5H_8O isomers and product ion interferences	10	10	10	III	
$C_6H_6O^+$	94.04	phenol	1	2	4	III	Phenol is most of signal in pre-burn
$C_6H_9O^+$	97.06	C_6H_8O isomers and product ion interferences	7	9	15	III	
$C_8H_7^+$	103.05	phenylacetylene	1	1	1	I	
$C_7H_6N^+$	104.05	benzonitrile	1	1	4	I	Very small cluster of peaks in post-burn
$C_8H_9^+$	105.07	styrene	1	1	1	I	
$C_7H_7O^+$	107.05	benzaldehyde	1	1	1	I	
$C_7H_9O^+$	109.06	C_7H_8O isomers	1	2	2	II	A lot of small peaks in pre- and post- but two major ones
$C_9H_9^+$	117.07	indene	0	1	1	I	Nothing eluted in background
$C_8H_7O^+$	119.05	benzofuran	2	1	1	I	
$C_9H_{11}^+$	119.09	C_9H_{10} isomers	6	7	4	III	Possible interferences

							from terpenoid fragments
$C_8H_9O^+$	121.06	C_8H_8O isomers	3	4	6	II	Two major peaks post- RT = 350 s (major product ion is dehydration product so maybe phenylacetaldehyde) and RT = 500 (maybe acetophenone)
$C_7H_7O_2^+$	123.04	$C_7H_6O_2$ isomers	2	2	3	II	No obvious product ion interferences
$C_8H_{11}O^+$	123.08	$C_8H_{10}O$ isomers and product ion interferences	7	6	5	III	Lot of smaller peaks
$C_{10}H_9$+	129.07	naphthalene	1	2	2	II	Major peak is naphthalene smaller peak may be azulene
C_9H_8N+	130.07	C_9H_7N isomers	0	0	0	II	Nothing eluted, but likely no product ion interferences
C_9H_7O+	131.05	C_9H_6O isomers	0	0	0	II	Nothing eluted, but likely no product ion interferences
$C_{10}H_{11}$+	131.09	$C_{10}H_{10}$ isomers and product ion interferences	5	6	8	III	
$C_9H_9O^+$	133.06	C_9H_8O isomers and product ion interferences	2	3	3	III	
$C_8H_7S^+$	135.03	benzothiophene	0	1	1	I	Nothing eluted in background

$C_8H_9O_2^+$	137.06	$C_8H_8O_2$	3	3	3	II	One major peak, no clear product ion interferences
$C_{11}H_{11}^+$	143.09	$C_{11}H_{10}$ isomers and product ion interferences	0	2	2	III	
$C_{10}H_9O^+$	145.06	$C_{10}H_8O$ isomers	0	1	3	II	likely no product ion interferences
$C_{11}H_{13}^+$	145.10	C11 aromatics	0	0	0	II	Nothing eluted, but likely no product ion interferences
$C_{12}H_{11}^+$	155.09	C12 aromatics	0	0	0	II	Nothing eluted, but likely no product ion interferences

*$C_6H_6O^+$ is the charge-transfer product ion of phenol. $C_6H_7O^+$ had major product ion interferences from benzene and other aromatics.
**Product ion interferences could be produced from dehydration reactions, water clustering, and/or fragmentation reactions between a NMOG and H_3O^+ or other contaminant reagent ions.

Table S2. NMOG chemical properties.

ID	Compound used for K_{OA} and vapor pressure estimation in EPA EPI Suite	Octanol-Air Partitioning Coefficient $\log(K_{OA})$*	Vapor Pressure (log (atm))**
acetonitrile	acetonitrile	2.31	-0.93
acrylonitrile	acrylonitrile	2.50	-0.84
pyrrole and C_4 nitriles	pyrrole	3.88	-1.46
propiolic acid and product ion interferences	propiolic acid	5.01	-2.43
benzene and product ion interferences	benzene	2.78	-0.90
pyridine and cyanobutadiene isomers	pyridine	4.00	-1.85

C_5H_8O isomers and product ion interferences	trans-2-methyl-2-butenal	3.46	-1.63
Phenol	phenol	6.33	-3.33
C_6H_8O isomers and product ion interferences	2,3-dimethylfuran	3.03	-1.47
phenylacetylene	phenylacetylene	4.14	-2.61
benzonitrile	benzonitrile	4.46	-2.65
Styrene	styrene	3.90	-2.07
benzaldehyde	benzaldehyde	4.44	-2.78
C_7H_8O isomers	anisole	4.01	-2.33
Indene	indene	4.11	-2.94
benzofuran	benzofuran	4.34	-3.59
C_9H_{10} isomers	α-methylstyrene	4.46	-2.60
C_8H_8O isomers	acetophenone	4.95	-3.45
$C_7H_6O_2$ isomers	benzoic acid	7.74	-6.00
$C_8H_{10}O$ isomers and product ion interferences	m-ethylphenol	6.99	-4.18
naphthalene	naphthalene	5.19	-3.41
C_9H_7N isomers	quinoline	6.20	-4.10
C_9H_6O isomers	phenylpropynal	6.58	-4.08
$C_{10}H_{10}$ isomers and product ion interferences	1,2-dihydronaphthalene	5.48	-3.27
C_9H_8O isomers and product ion interferences	acrylophenone	5.55	-3.50
benzothiophene	benzothiophene	4.92	-3.50
$C_8H_8O_2$	4-methoxybenzaldehyde	5.23	-4.40
$C_{11}H_{10}$ isomers and product ion interferences	1-methylnaphthalene	5.55	-3.75
$C_{10}H_8O$ isomers	2-phenylfuran	4.90	-4.30
C11 aromatics	1-pentynylbenzene	5.03	-3.60

C12 aromatics	biphenyl	6.15	-5.55

*Values from EPA Estimation Program Interface Suite v4.11.[3]

**Vapor pressure at 25 ˚C. Values from EPA Estimation Program Interface Suite v4.11.[3]

Table S3. NMOG E_r measured from the test house. Data in this table are used in Figures 2 through 5.

	E_r average (± standard deviation) (2σ)* ($\mu g\ h^{-1}$)					
**ID**	**Day -55**	**Day 2**	**Day 35**	**Day 60**	**Day 98**	**Day 105**
acetonitrile	3 (1)	38 (6)	5 (1)	4 (2)	3 (2)	6 (2)
acrylonitrile	2 (1)	63 (11)	5 (1)	4 (1)	5 (1)	4 (1)
pyrrole and C_4 nitriles	4 (1)	15 (2)	5 (1)	4 (1)	4 (1)	4 (1)
propiolic acid and product ion interferences	10 (4)	21 (1)	13 (2)	7 (1)	13 (2)	15 (2)
benzene and product ion interferences	8 (2)	52 (12)	6 (1)	4 (1)	5 (1)	5 (1)
Pyridine and cyanobutadiene isomers	1 (1)	10 (2)	2 (1)	1 (1)	2 (1)	2 (1)
C_5H_8O isomers and product ion interferences	14 (6)	47 (4)	51 (6)	40 (4)	35 (4)	45 (5)
phenol	52 (13)	136 (5)	71 (7)	65 (3)	59 (4)	68 (8)
C_6H_8O isomers and product ion interferences	14 (4)	45 (3)	34 (5)	27 (3)	27 (3)	27 (3)
phenylacetylene	3 (1)	130 (20)	4 (1)	4 (1)	5 (1)	3 (1)

benzonitrile	1 (1)	63 (6)	12 (2)	5 (1)	3 (1)	3 (1)
styrene	21 (4)	220 (20)	33 (3)	33 (2)	32 (2)	38 (2)
benzaldehyde	50 (9)	170 (10)	190 (20)	130 (10)	110 (5)	140 (8)
C_7H_8O isomers	Not detected	14 (1)	9 (2)	9 (1)	7 (1)	9 (1)
indene	2 (1)	100 (10)	6 (1)	3 (1)	5 (1)	3 (1)
benzofuran	0.1 (0.1)	15 (2)	1.6 (0.5)	0.5 (0.1)	1.7 (0.4)	0.7 (0.1)
C_9H_{10} isomers	2 (2)	81 (5)	220 (2)	25 (1)	25 (2)	27 (1)
C_8H_8O isomers	18 (8)	93 (4)	88 (8)	67 (3)	56 (3)	66 (5)
$C_7H_6O_2$ isomers	6 (4)	27 (2)	30 (4)	22 (3)	18 (2)	21 (2)
$C_8H_{10}O$ isomers and product ion interferences	4 (2)	13 (1)	8 (1)	7 (1)	7 (1)	7 (1)
naphthalene	7 (2)	280 (30)	48 (5)	20 (2)	20 (1)	14 (1)
C_9H_7N isomers	0.3 (0.1)	8.3 (0.5)	2.5 (0.3)	2.0 (0.1)	1.7 (0.1)	2.0 (0.1)
C_9H_6O isomers	0.3 (0.2)	8.1 (0.7)	1.0 (0.2)	0.4 (0.1)	0.6 (0.1)	0.3 (0.1)
$C_{10}H_{10}$ isomers and product ion interferences	1.3 (0.4)	17 (1)	2.9 (0.2)	1.9 (0.1)	3.2 (0.3)	2.6 (0.2)
C_9H_8O isomers and product ion interferences	0.9 (0.3)	9.1 (0.7)	3.5 (0.4)	3.3 (0.1)	3.0 (0.2)	2.7 (0.1)
benzothiophene	Not detected	12 (1)	4 (1)	4 (1)	5 (1)	6 (1)
$C_8H_8O_2$	3 (1)	11 (1)	11 (1)	10 (1)	8 (1)	10 (1)
$C_{11}H_{10}$ isomers and product ion interferences	6 (1)	43 (3)	16 (1)	13 (1)	12 (1)	12 (1)
$C_{10}H_8O$ isomers	0.3 (0.1)	7.0 (0.6)	2.1 (0.2)	1.5 (0.1)	1.7 (0.2)	1.0 (0.1)
C11 aromatics	0.9 (0.2)	7 (1)	1.5 (0.2)	1.6 (0.1)	2.8 (0.3)	1.8 (0.5)
C12 aromatics	0.5 (0.1)	28 (3)	7 (1)	4.7 (0.1)	3.5 (0.1)	3.2 (0.1)

*Averages and standard deviations are from the average day ± 2.5 days.

As shown in Figure 2 in the main text, NMOG E_r measured from both the NIST test house and Colorado house showed first-order decays. We fit exponential decay curves to available data and the off-gassing times determined from those fits are summarized below in Table S4. Additionally, Day 10 E_r values were estimated for the NIST test house from the exponential fits. Day 10 and Day 35 E_r values for the NIST test house and CO house are shown in Table S4.

Table S4. NMOG E_r off-gassing times (shown in Figure 2) and Day 10 and Day 35 E_r values (shown in Figure 5) for the NIST test house and the Colorado house. Missing values for the CO house indicate those ions were not reported by the Dresser, et al. study.

	E_r off-gassing times (days)		Day 10 E_r (± 30 %) (μg h^{-1})**		Day 35 E_r (± 30 %) (μg h^{-1})**	
ID*	NIST	CO House	NIST	CO House	NIST	CO House
acrylonitrile	< 10	6	30 (10)	50 (15)	5 (2)	9 (3)
pyrrole and C_4 nitriles	< 10	6	10 (3)	120 (40)	5 (2)	12 (4)
benzene and product ion interferences	<10	3	70 (20)	2260 (680)	6 (2)	340 (100)
phenol	15	8	100 (30)	1140 (340)	70 (20)	290 (90)
C_6H_8O isomers and product ion interferences	> 25	14	40 (10)	400 (100)	30 (10)	80 (20)
benzonitrile	17	3	40 (10)	90 (30)	10 (3)	4 (1)
styrene	< 10	3	90 (30)	1500 (500)	30 (10)	220 (70)
benzaldehyde	> 25	7	190 (60)	660 (220)	190 (60)	100 (30)
C_7H_8O isomers	> 25	4	10 (3)	860 (260)	9 (3)	100 (30)

indene	< 10	5	30 (10)	280 (80)	6 (2)	40 (10)
naphthalene	15	5	180 (50)	370 (110)	50 (15)	20 (5)
C12 aromatics	17	6	20 (5)	30 (10)	7 (2)	1 (0.3)

*IDs are from the categorization shown in Table S1 and thus apply most directly with test house measurements. Ions measured from Colorado house may have different uncertainty categorizations.

**The 30 % uncertainty shown here represents the uncertainty in determination of the λ propagated to E_r calculations.

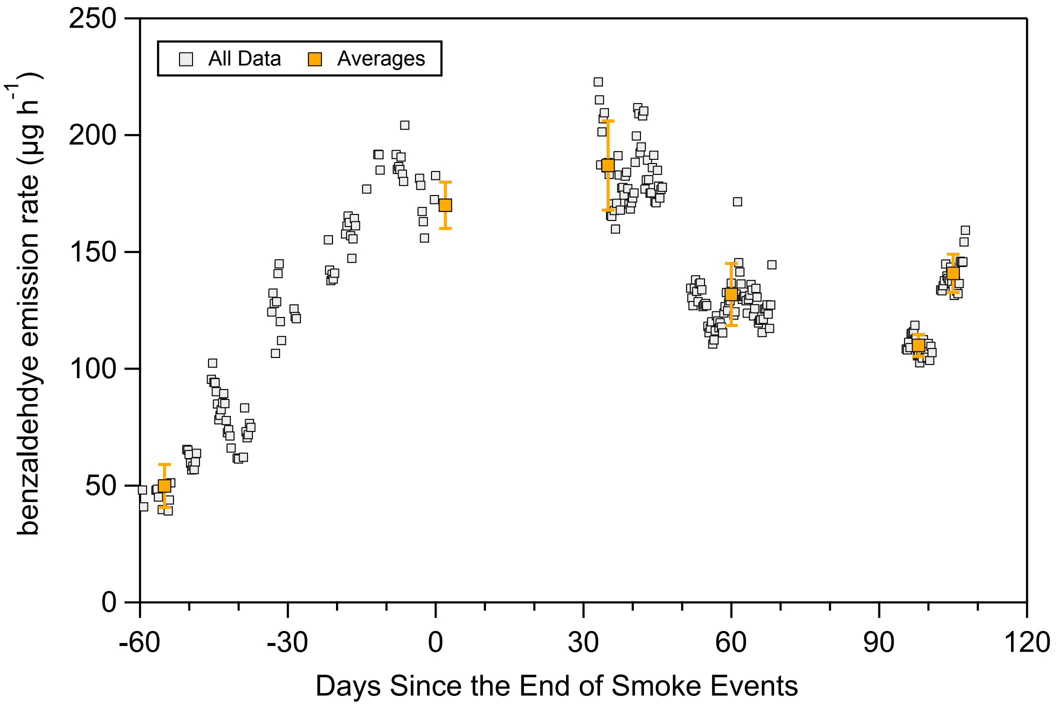

Figure S9. Benzaldehyde E_r during the measurement period. Benzaldehyde is an example of a NMOG that we could not calculate a first-order decay for from an exponential fit because the E_r increased going from Day 2 to Day 35. The error bars show the standard deviation (2σ) of the E_r averaged over five days.

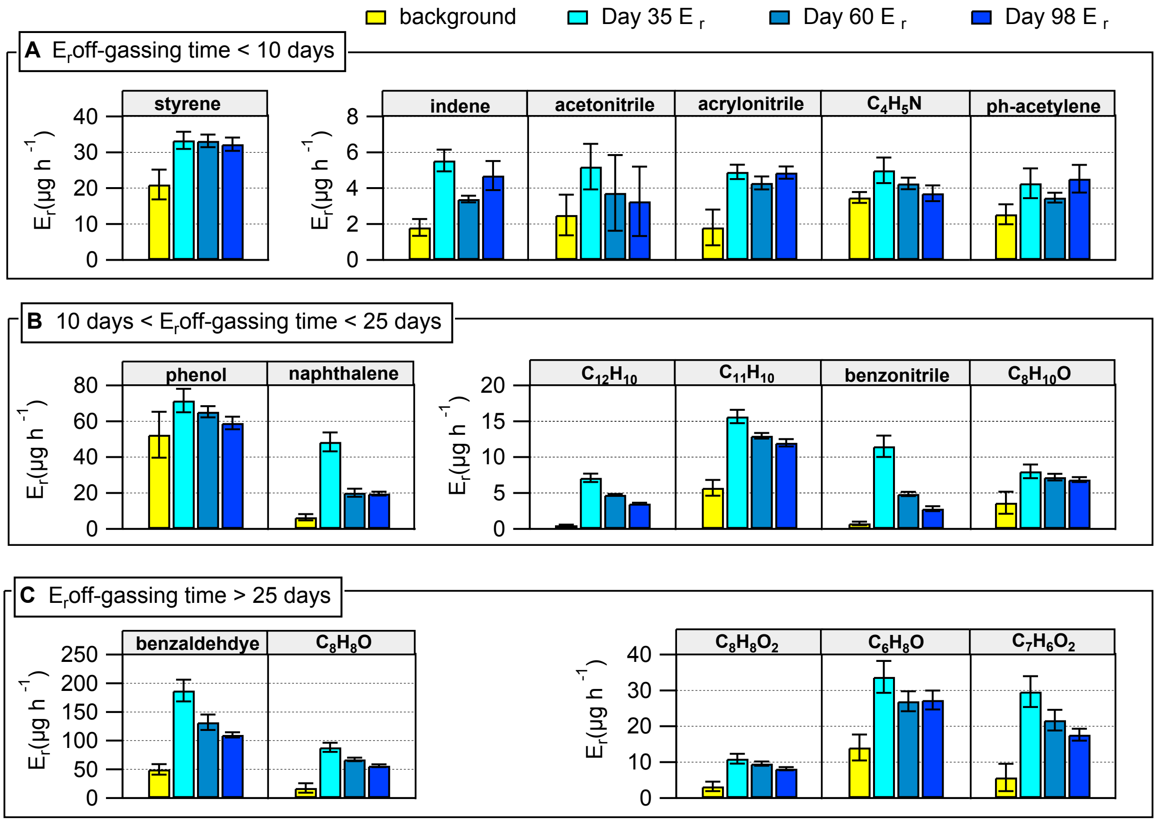

Figure S10. Average E_r for select NMOGs where the measured emission rate off-gassing time was (A) less than 10 days, (B) between 10 days and 25 days, and (C) more than 25 days. Colors of the bars correspond to the marker colors in Figure 2. Phenylacetylene is abbreviated as "ph-acetylene" in panel A. The error bars show the standard deviation (2σ) of the E_r averaged over five days.

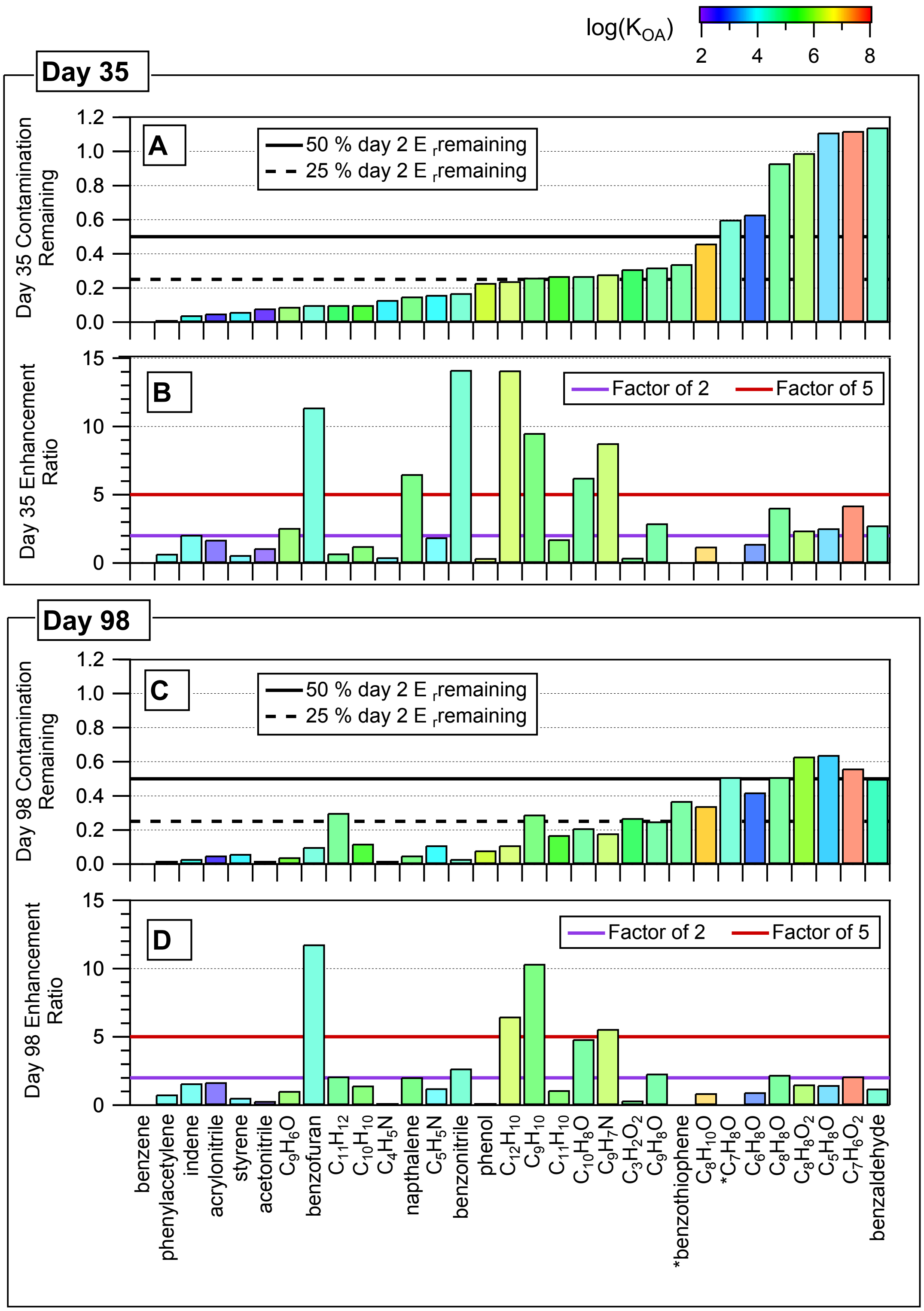

Figure S11. Corresponds to Figure 4 in the main text except all bars are colored by $\log(K_{OA})$. We did not observe any obvious trends between the fraction of contamination remaining and/or enhancement ratio with $\log(K_{OA})$.

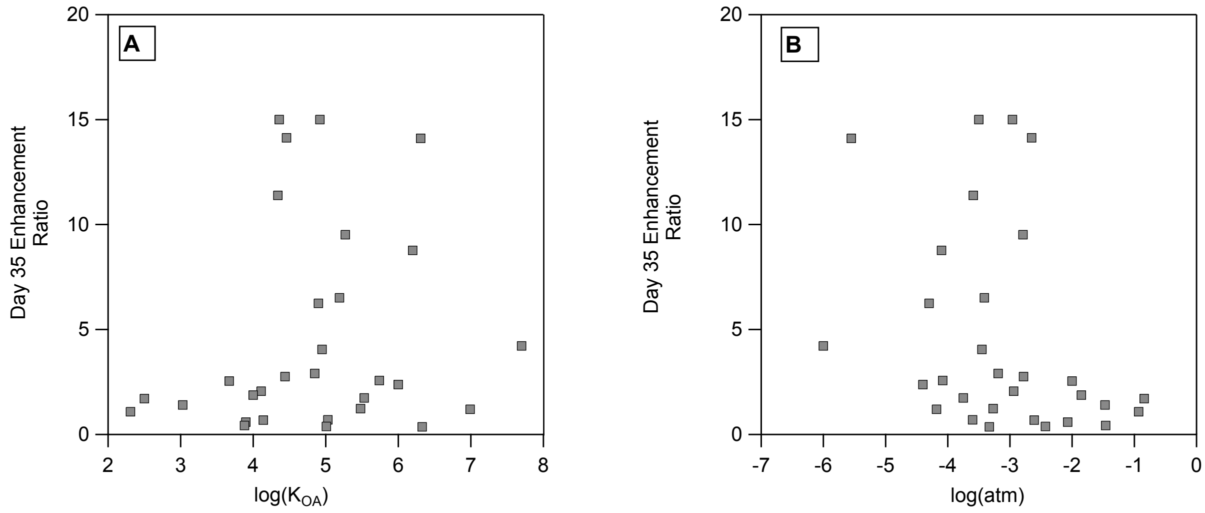

Figure S12. (A) Day 35 enhancement ratio plotted against log(K_{OA}). (B) Day 35 enhancement ratio plotted against log of the vapor pressure at 25 ˚C in units of atm. Values were acquired from the EPA EPI Suite v4.4.[3]

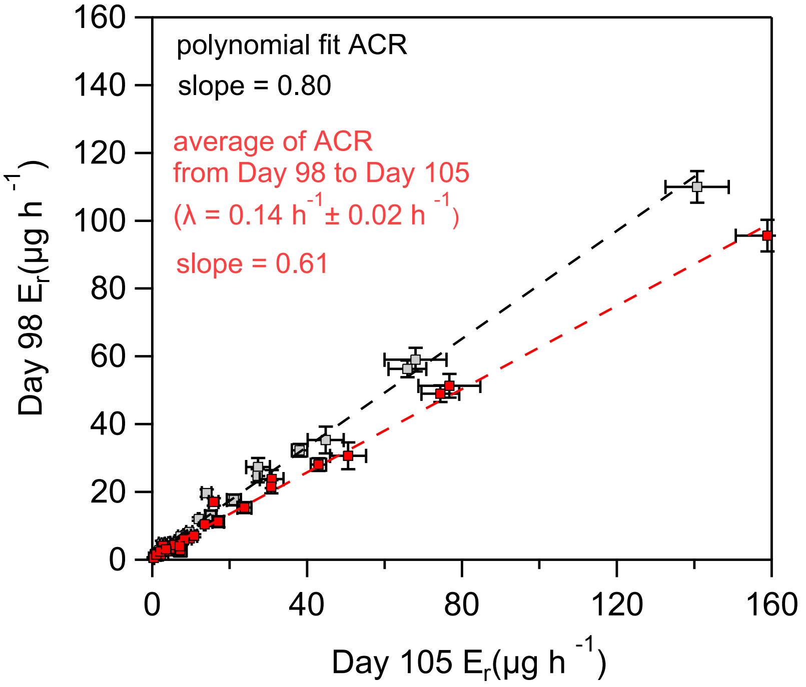

Figure S13. Day 98 E_r (several days prior to carpet removal) plotted against Day 105 E_r (several days after carpet removal) to demonstrate that most NMOG emissions decreased by approximately 20 %. The gray markers show the E_r values highlighted in the manuscript that were calculated using the polynomial fit ACR values. The error bars show the standard deviation

(2σ) of the E_r averaged over five days. We also show the relationship of Day 98 to Day 105 E_r with values calculated from using the average ACR of 0.14 h^{-1} ± 0.02 h^{-1} (red markers) to demonstrate the difference in using the polynomial fit ACR to an average ACR value.

References

(1) Karion, A.; Link, M. F.; Robertson, R.; Boyle, T.; Poppendieck, D. Methodology and uncertainty estimation for measurements of methane leakage in a manufactured house. *Atmospheric Measurement Techniques* **2024**, *17* (24), 7065-7075. DOI: 10.5194/amt-17-7065-2024.

(2) Zhang, Y.; Wang, Y.; Li, C.; Li, Y.; Yin, S.; Claflin, M. S.; Lerner, B. M.; Worsnop, D.; Wang, L. Interpretation of mass spectra by a Vocus proton transfer reaction mass spectrometer (PTR-MS) at an urban site: insights from gas-chromatographic pre-separation. *Atmospheric Measurement Techniques Discussions*, 2025.

(3) Estimation Programs Interface Suite™ for Microsoft® Windows, v 4.11. United States Environmental Protection Agency, Washington, DC, USA.
